# Checklist of aquatic and marshy Monocotyledons from the Araguaia River basin, Brazilian Cerrado

**DOI:** 10.3897/BDJ.4.e7085

**Published:** 2016-03-02

**Authors:** Adriana Oliveira, Claudia Bove

**Affiliations:** ‡ Universidade Federal do Rio de Janeiro, Rio de Janeiro, Brazil

**Keywords:** Aquatic plants, Brazilian savannah, Central Brazil flora, Lilianae

## Abstract

**Background:**

The Araguaia River basin runs through the states of Goiás, Mato Grosso, Tocantins, and Pará, covering 373,000 Km^2^, mostly within the Brazilian Cerrado. The region has a wide variety of wetlands. The climate is characterized by high temperatures and strongly seasonal precipitation. There are two well defined seasons: the dry season (winter-spring) and the rainy season (summer- fall). The Araguaia River basin is dominated by plinthosoils that are found in low flat areas, poorly drained and prone to flooding, yielding wetland habitats of high plant diversity. Since the 1970s, human activities have led to reduction in both the diversity and area of wetlands. The construction of the Belém-Brasília highway and hydroelectric dams, as well as the expansion of agricultural and mining activities, have had major impacts on the region.

**New information:**

The flora diversity data of the Araguaia River basin was developed through field work, herbarium research, and use of a database (*Species* Link). The resulting checklist of 162 aquatic and marshy monocotyledons from the Araguaia River basin represents 20 families and 50 genera. Cyperaceae (51 spp.), Poaceae (39 spp.), and Eriocaulaceae (16 spp.) are the most representative families. Life form analysis indicates that helophytes predominate (98 spp.; 60.5%). One hundred one species are native to tropical and/or subtropical America and twenty one are endemic to Brazil. Ninety-three species are new occurrences for the Araguaia River basin. Among them, three species are reported in the Brazilian Cerrado for the first time. This work contributes to the understanding of aquatic plant diversity in the Cerrado and other savanna-like vegetation physiognomies; environments and habitats poorly understood taxonomically and undercollected generally.

## Introduction

The Brazilian Cerrado is one of the most threatened ecosystems in Brazil and was ranked among the 34 most important biodiversity hotspots of the world (Mittermeier et al. 2004). In the first survey of the Cerrado flora, Mendonça et al. (1998) listed *ca.* 6,000 angiosperm species. After that, efforts to raise the knowledge on biodiversity for this area nearly doubled the number of species, with the monocotyledons (Lilianae) accounting for roughly 25% of the increase (Sano et al. 2008). The Araguaia River basin runs through the states of Goiás, Mato Grosso, Tocantins, and Pará; covering 373,000 Km^2^ (Lima et al. 2004), mostly within the Cerrado. The region is rich in wetlands like rivers, streams, temporary or permanent swamps and pools. The climate is characterized by high temperatures that reach 100.4 °F in September and October and strongly seasonal precipitation of 1750 to 2000 mm per year. There are two well- defined seasons: the dry season (July-November) and the rainy season (December-June) (IBGE 1989). This basin is dominated by plinthosoils, which are poorly drained and usually found in low flat areas prone to flooding (Resende et al. 1995), forming temporary or permanent swamps. The importance of wetland habitats ecologically and in terms of plant biodiversity globally has been emphasized by many authors (e.g. Gopal and Junk 2000, Thomaz and Cunha 2010, Paz and Bove 2011, Mendonça et al. 2012, Moura-Júnior et al. 2013). On the other hand their taxonomic diversity is rarely the focus of wetland studies (e.g. Pedralli et al. 997, Bove et al. 2003, Moreira et al. 2011, Contaifer et al. 2013, Sabino et al. 2015).

The first study to include aquatic plants in Brazil was carried out by Hoehne (1915) in the Cerrado and in the Amazon Forest. The same author published “Plantas Aquáticas” (Hoehne 1948), a pioneer study of aquatic flora from Brazil. Also, species from Pantanal have been studied (Pott and Pott 1997, Pott and Pott 2000). A checklist from the states of Mato Grosso and Mato Grosso do Sul (Dubs 1998) and the vascular flora of the Emas National Park, located in the Araguaia River basin (Batalha and Martins 2002) incidentally included some aquatic plants, though they did not survey aquatic ecosystems. In spite of the vast area encompassed by the Araguaia River basin and the broad diversity of wetland habitats, the aquatic and marshy flora of this region is little known. This work contributes to a better understanding of the aquatic environments in the Cerrado and other savanna-like vegetation physiognomies. Notably, herbarium collections are depauperate with respect to aquatic plants, reflecting both the difficulty of collecting in wetlands, as well as the scarcity of specialists in the taxonomic groups included. Generally, these plants are incidentally collected by terrestrial plant specialists and the quality of incidental collections may challenge correct identification. Thus, the real diversity of the aquatic flora from the Brazilian Cerrado will be better known through studies carried out by specialists focusing on aquatic habitats on the region. Our efforts to improve the knowledge of the aquatic flora from the Araguaia River basin have resulted in the discovery of new species (Koehler and Bove 2001, Oliveira et al. 2011) and on the better understanding of taxonomic groups such as the Alismatales (Koehler and Bove 2004) and Cyperaceae (Gil et al. 2007, Oliveira et al. 2011). As a result of an intensive search of collections and field work, we provide here an annotated list of aquatic and marshy monocots found in the Araguaia River basin. The results increase our knowledge of biodiversity in this basin and in the Cerrado. Species not previously reported to this area are highlighted. We also include images of the specific habitats and species from the Araguaia River basin.

## Materials and methods

**Figure 1.**
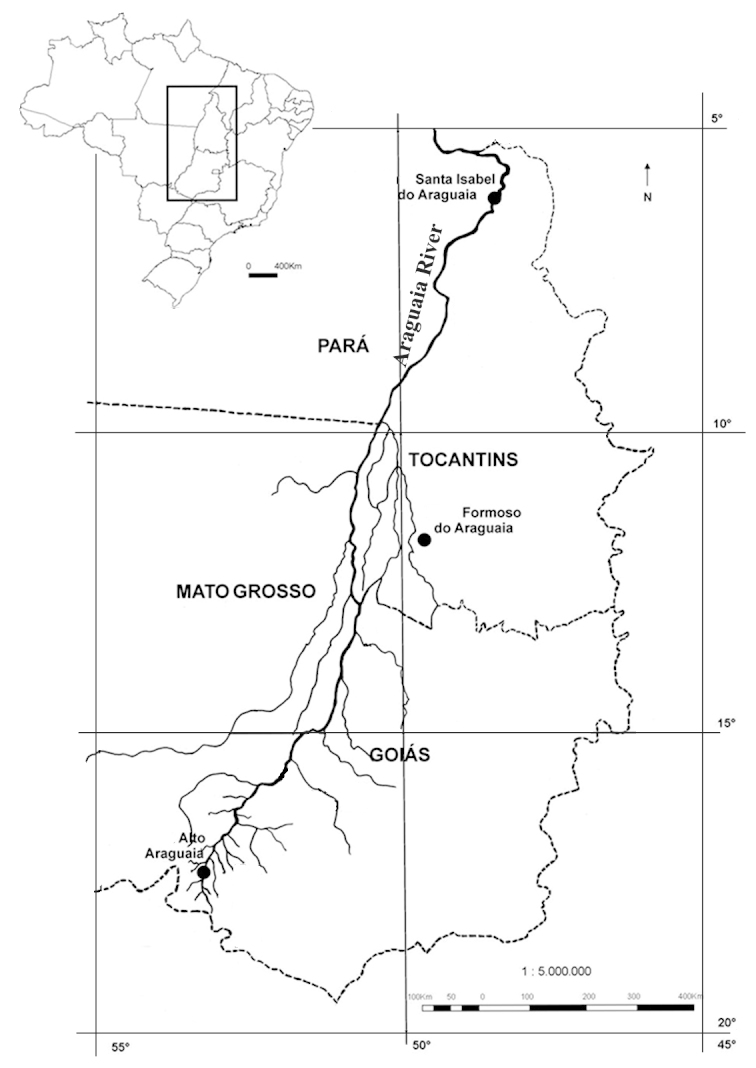
Araguaia River basin map (Gil et al. 2007, Oliveira et al. 2011).

Material for this study is from collections made between 5-20°S and 45–55°W, located approximately between the city of Alto Araguaia at the southern limit and the city of Formoso do Araguaia at the northern limit (Fig. 1). Collections were made randomly, primarily along state roads (MT-100, MT-126, MT-326, GO-164, GO-184, GO-173, GO-221, GO-324, GO-334, and GO-530) and federal roads (BR-070, BR-158, BR-242 and BR-251); in both, rainy and dry seasons (1997, 1999, 2004, 2006, and 2007). Part of this material has been used in previous studies (Gil et al. 2007, Koehler and Bove 2001, Koehler and Bove 2004, Oliveira and Bove 2011), which overlap some portion of the study area for the present study. Surveyed habitats included rivers, streams, seasonally and permanently flooded areas (Fig. 2). The specimens were dried following standard techniques (Fidalgo and Bononi 1989). Voucher materials were deposited in the herbarium of the Museu Nacional from the Federal University of Rio de Janeiro (R). Material from the following herbaria: R, RB, UB and UFG (http://sweetgum.nybg.org/science/ih/) were also analyzed. The search in the consulted herbaria was done analyzing all monocotyledonous material from the Araguaia River basin. The database SpeciesLink (2002) was consulted using the searching word “Araguaia”. Only those vouchers identified by experts in each family were added to the checklist. The data were compared to the previous bibliography on the Cerrado above mentioned and to the Lista de espécies da flora do Brasil (2010). The taxa were displayed in alphabetic order by family. The circumscription of families followed APG III (2009), the binomials and the geographic distribution is according the World Checklist of selected plant families (2013). The definition of aquatic plants adopted herein is sensu Cook 1996: “…whose photosynthetically active parts are permanently or, at least, for several months each year submerged in water or floating on the surface of water”. It includes the life form helophytes from the marshy areas as well the strictly aquatic species with their related life forms (submerged-fixed or submerged-free, floating-fixed or floating-free, and emergent).

## Checklists

### Checklist of hydrophylous Monocots from the Araguaia River basin

#### Echinodorus longipetalus

Mich.

##### Materials

- **Type status:** Other material. **Occurrence:** recordNumber: 158; recordedBy: C. P. Bove et al.; **Location:** country: Brazil; countryCode: BRA; stateProvince: Goiás; locality: Jussara-Aragarças road, 100 Km from Jussara; verbatimLatitude: 15°53'39.52"S; verbatimLongitude: 51°26'14.56"W; verbatimCoordinateSystem: degree minutes; **Event:** year: 1997; month: 5; day: 26; **Record Level:** institutionID: Museu Nacional Herbarium; institutionCode: R

#### Echinodorus subalatus

(Mart. ex Schult. f.) Griseb.

##### Materials

- **Type status:** Other material. **Occurrence:** recordNumber: 264; recordedBy: C. P. Bove et al.; **Location:** country: Brazil; countryCode: BRA; stateProvince: Mato Grosso; locality: Água Boa-Cocalinho road, 100 Km from Cocalinho; verbatimLatitude: 14°8'58.69"S; verbatimLongitude: 51°32'21.24"W; verbatimCoordinateSystem: degree minutes; **Event:** year: 1997; month: 10; day: 12; **Record Level:** institutionID: Museu Nacional Herbarium; institutionCode: R

##### Distribution

Britain to Ukraine, North Africa - WP.

#### Helanthium bolivianum

(Rusby) Lehtonen & Myllys

##### Materials

- **Type status:** Other material. **Occurrence:** recordNumber: 238; recordedBy: C. P. Bove et al.; **Location:** country: Brazil; countryCode: BRA; stateProvince: Goiás; locality: Jussara-Aragarças road, 100 Km from Jussara; verbatimLatitude: 15°53'39.52"S; verbatimLongitude: 51°26'14.56"W; verbatimCoordinateSystem: degree minutes; **Event:** year: 1997; month: 10; day: 9; **Record Level:** institutionID: Museu Nacional Herbarium; institutionCode: R

#### Helanthium tenellum

(Mart. ex Schult.f.) J.G.Sm.

##### Materials

- **Type status:** Other material. **Occurrence:** recordNumber: 161; recordedBy: C. P. Bove et al.; **Location:** country: Brazil; countryCode: BRA; stateProvince: Goiás; locality: Jussara-Aragarças road, 100 Km from Jussara; verbatimLatitude: 15°53'39.52"S; verbatimLongitude: 51°26'14.56"W; verbatimCoordinateSystem: degree minutes; **Event:** year: 1997; month: 5; day: 26; **Record Level:** institutionID: Museu Nacional Herbarium; institutionCode: R

##### Distribution

Holarctic, Pacific Is.

#### Hydrocleys nymphoides

(Willd.) Buchenau

##### Materials

- **Type status:** Other material. **Occurrence:** recordNumber: 606; recordedBy: C. P. Bove et al.; **Location:** country: Brazil; countryCode: BRA; stateProvince: Goiás; locality: Mozarlândia-Nova Crixás road, 18.6 Km from Mozarlândia; verbatimLatitude: 14°36'25.24"S; verbatimLongitude: 50°30'49.96"W; verbatimCoordinateSystem: degree minutes; **Event:** year: 1999; month: 11; day: 15; **Record Level:** institutionID: Museu Nacional Herbarium; institutionCode: R

#### Hydrocleys parviflora

Seub.

##### Materials

- **Type status:** Other material. **Occurrence:** recordNumber: 201; recordedBy: C. P. Bove et al.; **Location:** country: Brazil; countryCode: BRA; stateProvince: Goiás; locality: Jussara-Britânia road, 54 Km from Jussara; verbatimLatitude: 15°36'26.83"S; verbatimLongitude: 51°12'40.37"W; verbatimCoordinateSystem: degree minutes; **Event:** year: 1997; month: 5; day: 28; **Record Level:** institutionID: Museu Nacional Herbarium; institutionCode: R

##### Distribution

Mediterranean.

#### Limnocharis laforestii

Griseb.

##### Materials

- **Type status:** Other material. **Occurrence:** recordNumber: 205; recordedBy: C. P. Bove et al.; **Location:** country: Brazil; countryCode: BRA; stateProvince: Goiás; locality: Britânia-Aruanã road, 5 Km from Britânia; verbatimLatitude: 15°13'0.38"S; verbatimLongitude: 51°11'39.86"W; verbatimCoordinateSystem: degree minutes; **Event:** year: 1997; month: 5; day: 28; **Record Level:** institutionID: Museu Nacional Herbarium; institutionCode: R

##### Distribution

Holarctic.

#### Sagittaria guayanensis

Kunth

##### Materials

- **Type status:** Other material. **Occurrence:** recordNumber: 281; recordedBy: C. P. Bove et al.; **Location:** country: Brazil; countryCode: BRA; stateProvince: Goiás; locality: Araguapaz-Aruanã road, 11 Km from Aruanã; verbatimLatitude: 15°2'22.65"S; verbatimLongitude: 50°43'11.52"W; verbatimCoordinateSystem: degree minutes; **Event:** year: 1997; month: 10; day: 13; **Record Level:** institutionID: Museu Nacional Herbarium; institutionCode: R

##### Distribution

Cosmopolitan.

#### Sagittaria rhombifolia

Cham.

##### Materials

- **Type status:** Other material. **Occurrence:** recordNumber: 582; recordedBy: C. P. Bove et al.; **Location:** country: Brazil; countryCode: BRA; stateProvince: Goiás; locality: Jussara-Britânia road, 62.6 Km from Jussara, Pindaíba River; verbatimLatitude: 15°32'26.56"S; verbatimLongitude: 51°14'31.83"W; verbatimCoordinateSystem: degree minutes; **Event:** year: 1999; month: 11; day: 14; **Record Level:** institutionID: Museu Nacional Herbarium; institutionCode: R

##### Distribution

Holarctic.

#### Anthurium lindmanianum

Engl.

##### Materials

- **Type status:** Other material. **Occurrence:** recordNumber: 168; recordedBy: C. P. Bove et al.; **Location:** country: Brazil; countryCode: BRA; stateProvince: Goiás; locality: Jussara-Aragarças road, 100 Km from Jussara; verbatimLatitude: 15°53'39.52"S; verbatimLongitude: 51°26'14.56"W; verbatimCoordinateSystem: degree minutes; **Event:** year: 1997; month: 5; day: 26; **Record Level:** institutionID: Museu Nacional Herbarium; institutionCode: R

#### Dieffenbachia aglaonematifolia

Engl.

##### Materials

- **Type status:** Other material. **Occurrence:** recordNumber: 6516; recordedBy: J. A. Lombardi; **Location:** country: Brazil; countryCode: BRA; stateProvince: Mato Grosso; locality: MT-433, 11.7 Km from BR-158, Serra Nova Dourado, stream crossing road; verbatimLatitude: 12°09'30.0"S; verbatimLongitude: 51°36'49.0"W; verbatimCoordinateSystem: degree minutes; **Event:** year: 2006; month: 12; day: 17; **Record Level:** institutionID: Universidade Estadual Paulista Herbarium; institutionCode: HRCB

##### Distribution

Europe.

#### Dieffenbachia seguine

(Jacq.) Schott

##### Materials

- **Type status:** Other material. **Occurrence:** recordNumber: 615; recordedBy: C. P. Bove et al.; **Location:** country: Brazil; countryCode: BRA; stateProvince: Goiás; locality: GO- 334 road to Peixe, 8.8 Km from GO-164 road; verbatimLatitude: 14°52'27"S; verbatimLongitude: 50°30'16"W; verbatimCoordinateSystem: degree minutes; **Event:** year: 1999; month: 11; day: 16; **Record Level:** institutionID: Museu Nacional Herbarium; institutionCode: R

##### Distribution

Palearctic.

#### Gearum brasiliense

N.E.Br.

##### Materials

- **Type status:** Other material. **Occurrence:** recordNumber: 2241; recordedBy: J. Bogner; **Location:** country: Brazil; countryCode: BRA; stateProvince: Goiás; locality: Luiz Alves, 45 Km from São Miguel do Araguaia; verbatimLatitude: 13°14'02"S; verbatimLongitude: 50°33'42"W; verbatimCoordinateSystem: degree minutes; **Event:** year: 1996; month: 11; day: 4; **Record Level:** institutionID: Intituto Nacional de Pesquisas da Amazonia Herbarium; institutionCode: INPA

#### Philodendron imbe

Schott

##### Materials

- **Type status:** Other material. **Occurrence:** recordNumber: 14411; recordedBy: V. C. Souza; **Location:** country: Brazil; countryCode: BRA; stateProvince: Mato Grosso; locality: São Félix do Araguaia-Alto de Boa Vista road, 9 Km from São Félix do Araguaia; verbatimLatitude: 11°37'58.7"S; verbatimLongitude: 50°46'34.7"W; verbatimCoordinateSystem: degree minutes; **Event:** year: 1997; month: 3; day: 17; **Record Level:** institutionID: Universidade Estadual de Maringá Herbarium; institutionCode: HUEM

##### Distribution

Europe.

#### Philodendron quinquenervium

Miq.

##### Materials

- **Type status:** Other material. **Occurrence:** recordNumber: 1655; recordedBy: C. P. Bove et al.; **Location:** country: Brazil; countryCode: BRA; stateProvince: Goiás; locality: GO-164 road, 49.5 Km South of Nova Crixás; verbatimLatitude: 14°28'20.27"S; verbatimLongitude: 50°28'31.05"W; verbatimCoordinateSystem: degree minutes; **Event:** year: 2006; month: 4; day: 14; **Record Level:** institutionID: Museu Nacional Herbarium; institutionCode: R

##### Distribution

Italy

#### Urospatha sagittifolia

(Rudge) Schott

##### Materials

- **Type status:** Other material. **Occurrence:** recordNumber: 160; recordedBy: C. P. Bove et al.; **Location:** country: Brazil; countryCode: BRA; stateProvince: Goiás; locality: Jussara-Aragarças road, 100 Km from Jussara; verbatimLatitude: 15°53'39.52"S; verbatimLongitude: 51°26'14.56"W; verbatimCoordinateSystem: degree minutes; **Event:** year: 1997; month: 5; day: 26; **Record Level:** institutionID: Museu Nacional Herbarium; institutionCode: R

##### Distribution

Macedonia, Greece, Bulgaria.

#### Xanthosoma striatipes

(Kunth & C.D.Bouché) Madison

##### Materials

- **Type status:** Other material. **Occurrence:** recordNumber: 523; recordedBy: C. P. Bove et al.; **Location:** country: Brazil; countryCode: BRA; stateProvince: Goiás; locality: Jussara-Aragarças road, 100 Km from Jussara; verbatimLatitude: 15°53'39.52"S; verbatimLongitude: 51°26'14.56"W; verbatimCoordinateSystem: degree minutes; **Event:** year: 1999; month: 11; day: 11; **Record Level:** institutionID: Museu Nacional Herbarium; institutionCode: R

##### Distribution

Eastern Europe.

#### Desmoncus polyacanthos

Mart.

##### Materials

- **Type status:** Other material. **Occurrence:** recordNumber: 14405; recordedBy: V.C. Souza et al.; **Location:** country: Brazil; countryCode: BRA; stateProvince: Mato Grosso; locality: São Félix do Araguaia-Alto de Boa Vista road, 9 Km from São Félix do Araguaia; verbatimLatitude: 11°22'33.1"S; verbatimLongitude: 50°27'48.5"W; verbatimCoordinateSystem: degree minutes; **Event:** year: 1997; month: 3; day: 18; **Record Level:** institutionID: Universidade de São Paulo Herbarium; institutionCode: ESA

##### Distribution

Macedonia, Serbia, Montenegro.

#### Syagrus petraea

(Mart.) Becc.

##### Materials

- **Type status:** Other material. **Occurrence:** recordNumber: 14363; recordedBy: V.C. Souza et al.; **Location:** country: Brazil; countryCode: BRA; stateProvince: Mato Grosso; locality: São Félix do Araguaia, margin of Riozinho River; verbatimLatitude: 11°30'11.7"S; verbatimLongitude: 50°29'25.7"W; verbatimCoordinateSystem: degree minutes; **Event:** year: 1997; month: 3; day: 17; **Record Level:** institutionID: Universidade de São Paulo Herbarium; institutionCode: ESA

##### Distribution

Europe.

#### Apteria aphylla

(Nutt.) Barnhart ex Small

##### Materials

- **Type status:** Other material. **Occurrence:** recordNumber: 671; recordedBy: C. P. Bove et al.; **Location:** country: Brazil; countryCode: BRA; stateProvince: Goiás; locality: Caipônia-Iporá road (Km 54); verbatimLatitude: 16°43'45.15"S; verbatimLongitude: 51°29'18.64"W; verbatimCoordinateSystem: degree minutes; **Event:** year: 1999; month: 11; day: 19; **Record Level:** institutionID: Museu Nacional Herbarium; institutionCode: R

#### Burmannia capitata

(Walter ex J.F.Gmel.) Mart.

##### Materials

- **Type status:** Other material. **Occurrence:** recordNumber: 1809; recordedBy: C. P. Bove et al.; **Location:** country: Brazil; countryCode: BRA; stateProvince: Mato Grosso; locality: Alto Araguaia; verbatimLatitude: 17°18'64"S; verbatimLongitude: 53°13'08"W; verbatimCoordinateSystem: degree minutes; **Event:** year: 2007; month: 1; day: 10; **Record Level:** institutionID: Museu Nacional Herbarium; institutionCode: R

##### Distribution

Palearctic.

#### Burmannia flava

Mart.

##### Materials

- **Type status:** Other material. **Occurrence:** recordNumber: 597; recordedBy: C. P. Bove et al.; **Location:** country: Brazil; countryCode: BRA; stateProvince: Goiás; locality: Aruanã-Araguapaz road; verbatimLatitude: 14°58'00"S; verbatimLongitude: 50°52'00"W; verbatimCoordinateSystem: degree minutes; **Event:** year: 1999; month: 11; day: 19; **Record Level:** institutionID: Museu Nacional Herbarium; institutionCode: R

#### Canna indica

L.

##### Materials

- **Type status:** Other material. **Occurrence:** recordNumber: 8898; recordedBy: T. Plowman et al.; **Location:** country: Brazil; countryCode: BRA; stateProvince: Pará; locality: Santana do Araguaia, Redencão-Barreiras dos Campos road, PA-150, 100 Km South of Redencão; verbatimLatitude: 8°45'00.0"S; verbatimLongitude: 50°25'00.0"W; verbatimCoordinateSystem: degree minutes; **Event:** year: 1980; month: 2; day: 19; **Record Level:** institutionID: Missouri Botanical Garden Herbarium; institutionCode: MO

##### Distribution

Palearctic.

#### Commelina erecta

L.

##### Materials

- **Type status:** Other material. **Occurrence:** recordNumber: 17402; recordedBy: H. S. Irwin; **Location:** country: Brazil; countryCode: BRA; stateProvince: Mato Grosso; locality: 96 Km South of Xavantina; verbatimLatitude: 15°32'58.91"S; verbatimLongitude: 52°12'31.36"W; verbatimCoordinateSystem: degree minutes; **Event:** year: 1966; month: 6; day: 18; **Record Level:** institutionID: Smithsonian Institution; institutionCode: US

#### Commelina diffusa

Burm. f.

##### Materials

- **Type status:** Other material. **Occurrence:** recordNumber: 483; recordedBy: C. P. Bove et al.; **Location:** country: Brazil; countryCode: BRA; stateProvince: Goiás; locality: Jussara-Aragarças road; verbatimLatitude: 15°53'53"S; verbatimLongitude: 52°13'21"W; verbatimCoordinateSystem: degree minutes; **Event:** year: 1999; month: 11; day: 11; **Record Level:** institutionID: Museu Nacional Herbarium; institutionCode: R

##### Distribution

Holarctic.

#### Commelina obliqua

Vahl

##### Materials

- **Type status:** Other material. **Occurrence:** recordNumber: 9615; recordedBy: W. R. Anderson; **Location:** country: Brazil; countryCode: BRA; stateProvince: Goiás; locality: Caipônia, Serra dos Caiapós; verbatimLatitude: 16°57'15.57"S; verbatimLongitude: 51°48'34.36"W; verbatimCoordinateSystem: degree minutes; **Event:** year: 1973; month: 5; day: 2; **Record Level:** institutionID: Museu Nacional Herbarium; institutionCode: R

#### Costus spirales

(Jacq.) Roscoe

##### Materials

- **Type status:** Other material. **Occurrence:** recordNumber: 508; recordedBy: C. P. Bove et al.; **Location:** country: Brazil; countryCode: BRA; stateProvince: Goiás; locality: Jussara-Aragarças road, 97 Km from Jussara; verbatimLatitude: 15°52'58.60"S; verbatimLongitude: 51°27'18.43"W; verbatimCoordinateSystem: degree minutes; **Event:** year: 1999; month: 11; day: 11; **Record Level:** institutionID: Museu Nacional Herbarium; institutionCode: R

#### Ascolepis brasiliensis

(Kunth) Benth. ex C.B. Clarke

##### Materials

- **Type status:** Other material. **Occurrence:** recordNumber: 548; recordedBy: C. P. Bove et al.; **Location:** country: Brazil; countryCode: BRA; stateProvince: Goiás; locality: Jussara-Britânia road, 78.2 Km from Jussara; verbatimLatitude: 15°52'54.69"S; verbatimLongitude: 51°3'50.35"W; verbatimCoordinateSystem: degree minutes; **Event:** year: 1999; month: 11; day: 11; **Record Level:** institutionID: Museu Nacional Herbarium; institutionCode: R

#### Bulbostylis stenocarpa

Kük.

##### Materials

- **Type status:** Other material. **Occurrence:** recordNumber: 17073; recordedBy: H. S. Irwin; **Location:** country: Brazil; countryCode: BRA; stateProvince: Mato Grosso; locality: ca. 25 Km South of Xavantina, drainage of the Upper Araguaia River; verbatimLatitude: 14°52'12.0"S; verbatimLongitude: 52°19'12.0"W; verbatimCoordinateSystem: degree minutes; **Event:** year: 1996; month: 6; day: 13; **Record Level:** institutionID: Missouri Botanical Garden Herbarium; institutionCode: MO

#### Calyptrocarya glomerulata

(Brongn.) Urb.

##### Materials

- **Type status:** Other material. **Occurrence:** recordNumber: 650; recordedBy: C. P. Bove et al.; **Location:** country: Brazil; countryCode: BRA; stateProvince: Goiás; locality: Caiapônia-Piranhas road, 30 Km South of Caiapônia, Abóbora waterfall; verbatimLatitude: 16°47'12"S; verbatimLongitude: 51°43'16"W; verbatimCoordinateSystem: degree minutes; **Event:** year: 1999; month: 11; day: 18; **Record Level:** institutionID: Museu Nacional Herbarium; institutionCode: R

#### Cyperus diffusus

Vahl

##### Materials

- **Type status:** Other material. **Occurrence:** recordNumber: 14465; recordedBy: V.C. Souza et al.; **Location:** country: Brazil; countryCode: BRA; stateProvince: Mato Grosso; locality: São Félix do Araguaia; verbatimLatitude: 11°37'49"S; verbatimLongitude: 50°39'58"W; verbatimCoordinateSystem: degree minutes; **Event:** year: 1997; month: 3; day: 18; **Record Level:** institutionID: Universidade de São Paulo Herbarium; institutionCode: ESA

#### Cyperus digitatus

Roxb.

##### Materials

- **Type status:** Other material. **Occurrence:** recordNumber: 627; recordedBy: C. P. Bove et al.; **Location:** country: Brazil; countryCode: BRA; stateProvince: Goiás; locality: Peixe road, GO-334, 39.1Km from GO-164; verbatimLatitude: 14°56'34.22"S; verbatimLongitude: 50°14'33.60"W; verbatimCoordinateSystem: degree minutes; **Event:** year: 1999; month: 11; day: 16; **Record Level:** institutionID: Museu Nacional Herbarium; institutionCode: R

#### Cyperus giganteus

Vahl

##### Materials

- **Type status:** Other material. **Occurrence:** recordNumber: 7088; recordedBy: G. Windisch et al.; **Location:** country: Brazil; countryCode: BRA; stateProvince: Mato Grosso; locality: Cocalinho, MT 326 road; verbatimLatitude: 14°20'00.0"S; verbatimLongitude: 51°10'00.0"W; verbatimCoordinateSystem: degree minutes; **Event:** year: 1992; month: 10; day: 23; **Record Level:** institutionID: Universidade Federal do Rio Grande do Sul Herbarium; institutionCode: ICN

#### Cyperus haspan

L.

##### Materials

- **Type status:** Other material. **Occurrence:** recordNumber: 484; recordedBy: C. P. Bove et al.; **Location:** country: Brazil; countryCode: BRA; stateProvince: Goiás; locality: Jussara-Aragarças road, Água limpa River; verbatimLatitude: 15°52'10.31"S; verbatimLongitude: 51°32'58.63"W; verbatimCoordinateSystem: degree minutes; **Event:** year: 1999; month: 11; day: 11; **Record Level:** institutionID: Museu Nacional Herbarium; institutionCode: R

#### Cyperus odoratus

L.

##### Materials

- **Type status:** Other material. **Occurrence:** recordNumber: 529; recordedBy: C. P. Bove et al.; **Location:** country: Brazil; countryCode: BRA; stateProvince: Goiás; locality: Jussara-Aragarças road, 111 Km from Jussara; verbatimLatitude: 15°56'21.13"S; verbatimLongitude: 51°20'59.27"W; verbatimCoordinateSystem: degree minutes; **Event:** year: 1999; month: 11; day: 11; **Record Level:** institutionID: Museu Nacional Herbarium; institutionCode: R

#### Cyperus pohlii

(Nees) Steud.

##### Materials

- **Type status:** Other material. **Occurrence:** recordNumber: 9578; recordedBy: W. R. Anderson; **Location:** country: Brazil; countryCode: BRA; stateProvince: Goiás; locality: Serra do Caiapó, ca. 25 Km (straight line) SW of Caiapônia; verbatimLatitude: 17°06'25.6"S; verbatimLongitude: 51°49'00.0"W; verbatimCoordinateSystem: degree minutes; **Event:** year: 1973; month: 5; day: 1; **Record Level:** institutionID: The New York Botanical Garden Herbarium; institutionCode: NY

#### Cyperus schomburgkianus

Nees

##### Materials

- **Type status:** Other material. **Occurrence:** recordNumber: 87; recordedBy: M. A. Milaneze; **Location:** country: Brazil; countryCode: BRA; stateProvince: Tocantins; locality: Formoso do Araguaia, Formoso Duere road; verbatimLatitude: 11°47'10.25"S; verbatimLongitude: 49°31'15.88"W; verbatimCoordinateSystem: degree minutes; **Event:** year: 1994; month: 1; day: 14; **Record Level:** institutionID: Universidade Federal de Pernambuco Herbarium; institutionCode: UFP

#### Cyperus sphacelatus

Rottb.

##### Materials

- **Type status:** Other material. **Occurrence:** recordNumber: 8826; recordedBy: T. Plowman et al.; **Location:** country: Brazil; countryCode: BRA; stateProvince: Pará; locality: Conceição do Araguaia, ca. 20 Km West of Redenção; verbatimLatitude: 8°03'00.0"S; verbatimLongitude: 50°10'12.0"W; verbatimCoordinateSystem: degree minutes; **Event:** year: 1980; month: 2; day: 14; **Record Level:** institutionID: Missouri Botanical Garden Herbarium; institutionCode: MO

#### Cyperus tenuispica

Steud.

##### Materials

- **Type status:** Other material. **Occurrence:** recordNumber: 17095; recordedBy: H.S. Irwin et al.; **Location:** country: Brazil; countryCode: BRA; stateProvince: Mato Grosso; locality: ca. 20 Km. South of Xavantina, drainage of the Upper Araguaia River; verbatimLatitude: 14°49'12.0"S; verbatimLongitude: 52°19'12.0"W; verbatimCoordinateSystem: degree minutes; **Event:** year: 1966; month: 6; day: 13; **Record Level:** institutionID: Missouri Botanical Garden Herbarium; institutionCode: MO

#### Diplacrum capitatum

(Willd.) Boeck.

##### Materials

- **Type status:** Other material. **Occurrence:** recordNumber: 9097; recordedBy: T. Plowman et al.; **Location:** country: Brazil; countryCode: BRA; stateProvince: Pará; locality: 2 Km West of town, along highway PA-287; verbatimLatitude: 8°15'00.0"S; verbatimLongitude: 49°18'00.0"W; verbatimCoordinateSystem: degree minutes; **Event:** year: 1980; month: 2; day: 24; **Record Level:** institutionID: Intituto Nacional de Pesquisas da Amazonia Herbarium; institutionCode: INPA

#### Eleocharis acutangula

(Roxb.) Schult.

##### Materials

- **Type status:** Other material. **Occurrence:** recordNumber: 186; recordedBy: C. P. Bove et al.; **Location:** country: Brazil; countryCode: BRA; stateProvince: Goiás; locality: Jussara-Aragarças road, Km 100; verbatimLatitude: 14°49'10"S; verbatimLongitude: 50°58'36"W; verbatimCoordinateSystem: degree minutes; **Event:** year: 1997; month: 5; day: 26; **Record Level:** institutionID: Museu Nacional Herbarium; institutionCode: R

#### Eleocharis capillacea

Kunth

##### Materials

- **Type status:** Other material. **Occurrence:** recordNumber: 199b; recordedBy: C. P. Bove et al.; **Location:** country: Brazil; countryCode: BRA; stateProvince: Goiás; locality: Jussara-Aruanã road, Km 40; verbatimLatitude: 15°52'54.67"S; verbatimLongitude: 51°3'50.48"W; verbatimCoordinateSystem: degree minutes; **Event:** year: 1997; month: 5; day: 28; **Record Level:** institutionID: Museu Nacional Herbarium; institutionCode: R

#### Eleocharis filiculmis

Kunth

##### Materials

- **Type status:** Other material. **Occurrence:** recordNumber: 544; recordedBy: C. P. Bove et al.; **Location:** country: Brazil; countryCode: BRA; stateProvince: Goiás; locality: Jussara-Britânia road, cross Jacilândia, 78.2 Km from Jussara; verbatimLatitude: 15°52'54.69"S; verbatimLongitude: 51°3'50.35"W; verbatimCoordinateSystem: degree minutes; **Event:** year: 1999; month: 11; day: 12; **Record Level:** institutionID: Museu Nacional Herbarium; institutionCode: R

#### Eleocharis interstincta

(Vahl) Roem. & Schult.

##### Materials

- **Type status:** Other material. **Occurrence:** recordNumber: 291; recordedBy: C. P. Bove et al.; **Location:** country: Brazil; countryCode: BRA; stateProvince: Goiás; locality: Aruanã-Britânia road, Km 20; verbatimLatitude: 15°3'34.38"S; verbatimLongitude: 51°5'54.41"W; verbatimCoordinateSystem: degree minutes; **Event:** year: 1997; month: 10; day: 14; **Record Level:** institutionID: Museu Nacional Herbarium; institutionCode: R

#### Eleocharis minimavar.minima

Kunth

##### Materials

- **Type status:** Other material. **Occurrence:** recordNumber: 17099; recordedBy: H.S. Irwin et al.; **Location:** country: Brazil; countryCode: BRA; stateProvince: Mato Grosso; locality: ca 20 Km South of Xavantina, drainage of the upper Araguaia River; verbatimLatitude: 14°49'59.62"S; verbatimLongitude: 52°17'45.74"W; verbatimCoordinateSystem: degree minutes; **Event:** year: 1966; month: 6; day: 13; **Record Level:** institutionID: Missouri Botanical Garden Herbarium; institutionCode: MO

#### Eleocharis minimavar.bicolor

(Chapm.) Svenson

##### Materials

- **Type status:** Other material. **Occurrence:** recordNumber: 592; recordedBy: C. P. Bove et al.; **Location:** country: Brazil; countryCode: BRA; stateProvince: Goiás; locality: Aruanã-Araguapaz road, 22.4 Km from Aruanã; verbatimLatitude: 14°58'48.24"S; verbatimLongitude: 50°53'11.38"W; verbatimCoordinateSystem: degree minutes; **Event:** year: 1999; month: 11; day: 15; **Record Level:** institutionID: Museu Nacional Herbarium; institutionCode: R

#### Eleocharis nana

Kunth

##### Materials

- **Type status:** Other material. **Occurrence:** recordNumber: 530; recordedBy: S. Moore; **Location:** country: Brazil; countryCode: BRA; stateProvince: Mato Grosso; locality: Santa Cruz do Xingu; verbatimLatitude: 10°9'19.14"S; verbatimLongitude: 52°23'30.88"W; verbatimCoordinateSystem: degree minutes; **Event:** year: 1891-1892; **Record Level:** institutionID: Museu Nacional Herbarium; institutionCode: R

#### Eleocharis nudipes

(Kunth) Palla

##### Materials

- **Type status:** Other material. **Occurrence:** recordNumber: 185; recordedBy: C. P. Bove et al.; **Location:** country: Brazil; countryCode: BRA; stateProvince: Goiás; locality: Jussara-Aragarças road, Km 100; verbatimLatitude: 14°49'10"S; verbatimLongitude: 50°58'36"W; verbatimCoordinateSystem: degree minutes; **Event:** year: 1997; month: 5; day: 26; **Record Level:** institutionID: Museu Nacional Herbarium; institutionCode: R

#### Eleocharis plicarhachis

(Griseb.) Svenson

##### Materials

- **Type status:** Other material. **Occurrence:** recordNumber: 230b; recordedBy: C. P. Bove et al.; **Location:** country: Brazil; countryCode: BRA; stateProvince: Goiás; locality: Jussara-Aragarças road, Km 100; verbatimLatitude: 14°49'10"S; verbatimLongitude: 50°58'36"W; verbatimCoordinateSystem: degree minutes; **Event:** year: 1997; month: 10; day: 9; **Record Level:** institutionID: Museu Nacional Herbarium; institutionCode: R

#### Eleocharis retroflexa

(Poir.) Urb.

##### Materials

- **Type status:** Other material. **Occurrence:** recordNumber: 1649; recordedBy: C. P. Bove et al.; **Location:** country: Brazil; countryCode: BRA; stateProvince: Goiás; locality: Aruanã-Araguapaz road; verbatimLatitude: 14°49'10"S; verbatimLongitude: 50°58'36"W; verbatimCoordinateSystem: degree minutes; **Event:** year: 2006; month: 4; day: 13; **Record Level:** institutionID: Museu Nacional Herbarium; institutionCode: R

#### Eleocharis sellowiana

Kunth

##### Materials

- **Type status:** Other material. **Occurrence:** recordNumber: 1326; recordedBy: C. P. Bove et al.; **Location:** country: Brazil; countryCode: BRA; stateProvince: Mato Grosso; locality: Alto Araguaia, Sapo stream; verbatimLatitude: 17°18'64"S; verbatimLongitude: 53°13'08"W; verbatimCoordinateSystem: degree minutes; **Event:** year: 2004; month: 1; day: 14; **Record Level:** institutionID: Museu Nacional Herbarium; institutionCode: R

#### Fimbristylis aestivalis

(Retz.) Vahl

##### Materials

- **Type status:** Other material. **Occurrence:** recordNumber: 608 a; recordedBy: C. P. Bove et al.; **Location:** country: Brazil; countryCode: BRA; stateProvince: Goiás; locality: Mozarlândia-Nova Crixás road; verbatimLatitude: 14°27'00"S; verbatimLongitude: 50°27'00"W; verbatimCoordinateSystem: degree minutes; **Event:** year: 1999; month: 11; day: 15; **Record Level:** institutionID: Museu Nacional Herbarium; institutionCode: R

#### Fimbristylis dichotoma

(L.) Vahl

##### Materials

- **Type status:** Other material. **Occurrence:** recordNumber: 510; recordedBy: C. P. Bove et al.; **Location:** country: Brazil; countryCode: BRA; stateProvince: Goiás; locality: Jussara-Aragarças road, 97 Km from Jussara; verbatimLatitude: 15°52'58.60"S; verbatimLongitude: 51°27'18.43"W; verbatimCoordinateSystem: degree minutes; **Event:** year: 1999; month: 11; day: 11; **Record Level:** institutionID: Museu Nacional Herbarium; institutionCode: R

#### Fimbristylis quinquangularis

(Vahl) Kunth

##### Materials

- **Type status:** Other material. **Occurrence:** recordNumber: 1668; recordedBy: C. P. Bove et al.; **Location:** country: Brazil; countryCode: BRA; stateProvince: Tocantins; locality: BR 242, Formoso do Araguaia-São João do Javaés road; verbatimLatitude: 11°47'36"S; verbatimLongitude: 49°43'31"W; verbatimCoordinateSystem: degree minutes; **Event:** year: 2006; month: 4; day: 15; **Record Level:** institutionID: Museu Nacional Herbarium; institutionCode: R

#### Fuirena umbellata

Rottb.

##### Materials

- **Type status:** Other material. **Occurrence:** recordNumber: 540; recordedBy: C. P. Bove et al.; **Location:** country: Brazil; countryCode: BRA; stateProvince: Goiás; locality: Jussara-Britânia road cross Jacilândia, 78.2 Km from Jussara; verbatimLatitude: 15°52'54.69"S; verbatimLongitude: 51°3'50.35"W; verbatimCoordinateSystem: degree minutes; **Event:** year: 1999; month: 11; day: 12; **Record Level:** institutionID: Museu Nacional Herbarium; institutionCode: R

#### Kyllinga brevifolia

Rottb.

##### Materials

- **Type status:** Other material. **Occurrence:** recordNumber: 9111; recordedBy: T. Plowman et al.; **Location:** country: Brazil; countryCode: BRA; stateProvince: Pará; locality: Conceição do Araguaia, 2 Km West of town along highway PA-287; verbatimLatitude: 8°15'00.0"S; verbatimLongitude: 49°18'00.0"W; verbatimCoordinateSystem: degree minutes; **Event:** year: 1980; month: 2; day: 24; **Record Level:** institutionID: Intituto Nacional de Pesquisas da Amazonia Herbarium; institutionCode: INPA

#### Kyllinga odorata

Vahl

##### Materials

- **Type status:** Other material. **Occurrence:** recordNumber: 14254; recordedBy: V. C. Souza et al.; **Location:** country: Brazil; countryCode: BRA; stateProvince: Mato Grosso; locality: São Félix do Araguaia; verbatimLatitude: 11°37'49"S; verbatimLongitude: 50°39'58"W; verbatimCoordinateSystem: degree minutes; **Event:** year: 1997; month: 3; day: 16; **Record Level:** institutionID: Universidade de São Paulo Herbarium; institutionCode: ESA

#### Kyllinga vaginata

Lam.

##### Materials

- **Type status:** Other material. **Occurrence:** recordNumber: 6518; recordedBy: J. A. Lombardi; **Location:** country: Brazil; countryCode: BRA; stateProvince: Mato Grosso; locality: Bom Jesus do Araguia, MT-433, Serra Nova Dourada, 11.7 Km to BR-158; verbatimLatitude: 12°21'54.7"S; verbatimLongitude: 51°44'24.7"W; verbatimCoordinateSystem: degree minutes; **Event:** year: 2006; month: 12; day: 17; **Record Level:** institutionID: Universidade Federal de Minas Gerais Herbarium; institutionCode: BHCB

#### Lipocarpha chinensis

(Osbeck) J.Kern

##### Materials

- **Type status:** Other material. **Occurrence:** recordNumber: 1640; recordedBy: C. P. Bove et al.; **Location:** country: Brazil; countryCode: BRA; stateProvince: Goiás; locality: Palestina, GO-221, Km 52; verbatimLatitude: 16°41'50.12"S; verbatimLongitude: 51°25'9.81"W; verbatimCoordinateSystem: degree minutes; **Event:** year: 2006; month: 4; day: 11; **Record Level:** institutionID: Museu Nacional Herbarium; institutionCode: R

#### Pycreus lanceolatus

(Poir.) C.B.Clark

##### Materials

- **Type status:** Other material. **Occurrence:** recordNumber: 1830; recordedBy: C. P. Bove et al.; **Location:** country: Brazil; countryCode: BRA; stateProvince: Goiás; locality: Mozarlândia, GO-164, 16.3 Km North from Mozarlânida; verbatimLatitude: 14°28'27.5"S; verbatimLongitude: 50°28'21.7"W; verbatimCoordinateSystem: degree minutes; **Event:** year: 2007; month: I; day: 19; **Record Level:** institutionID: Museu Nacional Herbarium; institutionCode: R

#### Pycreus unioloides

(R.Br.) Urb.

##### Materials

- **Type status:** Other material. **Occurrence:** recordNumber: 518; recordedBy: C. P. Bove et al.; **Location:** country: Brazil; countryCode: BRA; stateProvince: Goiás; locality: Jussara-Aragarças road, Km 100; verbatimLatitude: 14°49'10"S; verbatimLongitude: 50°58'36"W; verbatimCoordinateSystem: degree minutes; **Event:** year: 1999; month: 11; day: 11; **Record Level:** institutionID: Museu Nacional Herbarium; institutionCode: R

#### Rhynchospora armerioides

J.Presl & C.Presl.

##### Materials

- **Type status:** Other material. **Occurrence:** recordNumber: 16971; recordedBy: H. S. Irwin et al.; **Location:** country: Brazil; countryCode: BRA; stateProvince: Mato Grosso; locality: ca. 30 Km South of Xavantina, drainage of the Upper Araguaia River; verbatimLatitude: 14°55'12.0"S; verbatimLongitude: 52°19'12.0"W; verbatimCoordinateSystem: degree minutes; **Event:** year: 1966; month: 6; day: 11; **Record Level:** institutionID: Missouri Botanical Garden Herbarium; institutionCode: MO

#### Rhynchospora barbata

(Vahl) Kunth

##### Materials

- **Type status:** Other material. **Occurrence:** recordNumber: 218b; recordedBy: C. P. Bove et al.; **Location:** country: Brazil; countryCode: BRA; stateProvince: Goiás; locality: Aruanã-Araguapaz road, Km 4 from Aruanã; verbatimLatitude: 14°56'6.55"S; verbatimLongitude: 51°3'36.79"W; verbatimCoordinateSystem: degree minutes; **Event:** year: 1997; month: 5; day: 29; **Record Level:** institutionID: Museu Nacional Herbarium; institutionCode: R

#### Rhynchospora brevirostris

Griseb.

##### Materials

- **Type status:** Other material. **Occurrence:** recordNumber: 6931a; recordedBy: F. C. Hoehne; **Location:** country: Brazil; countryCode: BRA; stateProvince: Mato Grosso; locality: Manso River; verbatimLatitude: 14°41'34.73"S; verbatimLongitude: 56°15'49.18"W; verbatimCoordinateSystem: degree minutes; **Event:** year: 1911; month: 4; **Record Level:** institutionID: Museu Nacional Herbarium; institutionCode: R

#### Rhynchospora corymbosa

(L.) Britton

##### Materials

- **Type status:** Other material. **Occurrence:** recordNumber: 614; recordedBy: C. P. Bove et al.; **Location:** country: Brazil; countryCode: BRA; stateProvince: Goiás; locality: Mozarlância-Nova Crixás road; verbatimLatitude: 14°27'00"S; verbatimLongitude: 50°27'00"W; verbatimCoordinateSystem: degree minutes; **Event:** year: 1999; month: 11; day: 15; **Record Level:** institutionID: Museu Nacional Herbarium; institutionCode: R

#### Rhynchospora divaricata

(Ham.) M.T.Strong

##### Materials

- **Type status:** Other material. **Occurrence:** recordNumber: 14170; recordedBy: V. C. Souza et al.; **Location:** country: Brazil; countryCode: BRA; stateProvince: Mato Grosso; locality: São Félix do Araguaia-Alto da Boa Vista road; verbatimLatitude: 11°36'02.9"S; verbatimLongitude: 51°04'57.0"W; verbatimCoordinateSystem: degree minutes; **Event:** year: 1997; month: 3; day: 15; **Record Level:** institutionID: Universidade de São Paulo Herbarium; institutionCode: ESA

#### Rhynchospora exaltata

Kunth

##### Materials

- **Type status:** Other material. **Occurrence:** recordNumber: 4419; recordedBy: J. A. Ratter; **Location:** country: Brazil; countryCode: BRA; stateProvince: Tocantins; locality: Ilha do Bananal, National Park of Araguaia; verbatimLatitude: 10°55'12.95"S; verbatimLongitude: 50°11'0.37"W; verbatimCoordinateSystem: degree minutes; **Event:** year: 1980; month: 9; day: 14; **Record Level:** institutionCode: UEC

#### Rhynchospora filiformis

Vahl

##### Materials

- **Type status:** Other material. **Occurrence:** recordNumber: 14808; recordedBy: V. C. Souza et al.; **Location:** country: Brazil; countryCode: BRA; stateProvince: Mato Grosso; locality: São Félix do Araguaia, Pontinópolis town-Serra dos Magalhães road; verbatimLatitude: 11°33'39.4"S; verbatimLongitude: 51°13'00.7"W; verbatimCoordinateSystem: degree minutes; **Event:** year: 1997; month: 3; day: 21; **Record Level:** institutionID: Universidade de São Paulo Herbarium; institutionCode: ESA

#### Rhynchospora globosa

(Kunth) Roem. & Schult.

##### Materials

- **Type status:** Other material. **Occurrence:** recordNumber: 9652; recordedBy: W. R. Anderson; **Location:** country: Brazil; countryCode: BRA; stateProvince: Goiás; locality: 12 Km South of Caiapônia; verbatimLatitude: 17°4'6.52"S; verbatimLongitude: 51°51'39.77"W; verbatimCoordinateSystem: degree minutes; **Event:** year: 1973; month: 5; day: 2; **Record Level:** institutionID: Museu Nacional Herbarium; institutionCode: R

#### Rhynchospora hassleri

C.B.Clarke

##### Materials

- **Type status:** Other material. **Occurrence:** recordNumber: 9087; recordedBy: T. Plowman et al.; **Location:** country: Brazil; countryCode: BRA; stateProvince: Pará; locality: Conceição do Araguaia, 2 Km West of town, along highway PA-287; verbatimLatitude: 8°15'00.0"S; verbatimLongitude: 49°18'00.0"W; verbatimCoordinateSystem: degree minutes; **Event:** year: 1980; month: 2; day: 24; **Record Level:** institutionID: Intituto Nacional de Pesquisas da Amazonia Herbarium; institutionCode: INPA

#### Rhynchospora rugosa

(Vahl) Gale

##### Materials

- **Type status:** Other material. **Occurrence:** recordNumber: 17969; recordedBy: H. S. Irwin et al.; **Location:** country: Brazil; countryCode: BRA; stateProvince: Goiás; locality: Caiapônia to Jataí road, ca. 30 Km South of Caiapônia, Serra do Caiapó; verbatimLatitude: 17°09'00.0"S; verbatimLongitude: 51°49'12.0"W; verbatimCoordinateSystem: degree minutes; **Event:** year: 1966; month: 6; day: 29; **Record Level:** institutionID: Universidade de Brasília Herbarium; institutionCode: UB

#### Rhynchospora tenuis

Link.

##### Materials

- **Type status:** Other material. **Occurrence:** recordNumber: 17309; recordedBy: H.S. Irwin et al.; **Location:** country: Brazil; countryCode: BRA; stateProvince: Mato Grosso; locality: ca. 75 Km South of Xavantina, drainage of the upper Araguaia River, Serra Azul; verbatimLatitude: 15°17'14.74"S; verbatimLongitude: 52°10'56.78"W; verbatimCoordinateSystem: degree minutes; **Event:** year: 1966; month: 6; day: 17; **Record Level:** institutionID: Missouri Botanical Garden Herbarium; institutionCode: MO

#### Rhynchospora trispicata

(Nees) Schrad. ex Steud.

##### Materials

- **Type status:** Other material. **Occurrence:** recordNumber: 1658; recordedBy: C. P. Bove et al.; **Location:** country: Brazil; countryCode: BRA; stateProvince: Goiás; locality: GO-164, 49.5Km, South from Nova Crixás; verbatimLatitude: 14°28'20.27"S; verbatimLongitude: 50°28'31.05"W; verbatimCoordinateSystem: degree minutes; **Event:** year: 2006; month: 4; day: 14; **Record Level:** institutionID: Museu Nacional Herbarium; institutionCode: R

#### Rhynchospora aff. unisetosa

T. Koyama

##### Materials

- **Type status:** Other material. **Occurrence:** recordNumber: 14809; recordedBy: V. C. Souza et al.; **Location:** country: Brazil; countryCode: BRA; stateProvince: Mato Grosso; locality: São Félix do Araguaia, Pontinópolis town -Serra dos Magalhães road; verbatimLatitude: 11°20'02.0"S; verbatimLongitude: 51°07'48.0"W; verbatimCoordinateSystem: degree minutes; **Event:** year: 1997; month: 3; day: 21; **Record Level:** institutionID: Universidade de São Paulo Herbarium; institutionCode: ESA

#### Rhynchospora velutina

(Kunth) Boeck.

##### Materials

- **Type status:** Other material. **Occurrence:** recordNumber: 6106; recordedBy: A. Rizzo; **Location:** country: Brazil; countryCode: BRA; stateProvince: Goiás; locality: Amorinopólis to Verde River road, 40 Km from Amorinopólis, Serra dos Caiapós; verbatimLatitude: 16°37'16"S; verbatimLongitude: 51°5'41"W; verbatimCoordinateSystem: degree minutes; **Event:** year: 1971; month: 2; day: 20; **Record Level:** institutionID: Universidade Federal de Goiás Herbarium; institutionCode: UFG

#### Scleria gaertneri

Raddi

##### Materials

- **Type status:** Other material. **Occurrence:** recordNumber: 1653; recordedBy: C. P. Bove et al.; **Location:** country: Brazil; countryCode: BRA; stateProvince: Goiás; locality: Aruanã-Canga; verbatimLatitude: 14°49'10"S; verbatimLongitude: 50°58'36.4"W; verbatimCoordinateSystem: degree minutes; **Event:** year: 2006; month: 4; day: 13; **Record Level:** institutionID: Museu Nacional Herbarium; institutionCode: R

#### Scleria microcarpa

Nees ex Kunth

##### Materials

- **Type status:** Other material. **Occurrence:** recordNumber: 9086; recordedBy: T. Plowman et al.; **Location:** country: Brazil; countryCode: BRA; stateProvince: Pará; locality: Conceição do Araguaia, 2 Km West of town, along highway PA-287; verbatimLatitude: 8°15'00.0"S; verbatimLongitude: 49°18'00.0"W; verbatimCoordinateSystem: degree minutes; **Event:** year: 1980; month: 2; day: 24; **Record Level:** institutionID: Intituto Nacional de Pesquisas da Amazonia Herbarium; institutionCode: INPA

#### Scleria mitis

P.J.Bergius

##### Materials

- **Type status:** Other material. **Occurrence:** recordNumber: 564; recordedBy: C. P. Bove et al.; **Location:** country: Brazil; countryCode: BRA; stateProvince: Goiás; locality: Jussara-Britânia road, cross Jacilândia, 25 Km from Jussara; verbatimLatitude: 15°52'55.13"S; verbatimLongitude: 51°3'50.43"W; verbatimCoordinateSystem: degree minutes; **Event:** year: 1999; month: 11; day: 12; **Record Level:** institutionID: Museu Nacional Herbarium; institutionCode: R

#### Eriocaulon araguaiense

A.Oliveira & C.P.Bove

##### Materials

- **Type status:** Other material. **Occurrence:** recordNumber: 596; recordedBy: C. P. Bove et al.; **Location:** country: Brazil; countryCode: BRA; stateProvince: Goiás; locality: Aruanã-Araguapaz road, Km 22; verbatimLatitude: 14°58'48.12"S; verbatimLongitude: 50°53'11.62"W; verbatimCoordinateSystem: degree minutes; **Event:** year: 1999; month: 11; day: 15; **Record Level:** institutionID: Museu Nacional Herbarium; institutionCode: R

#### Eriocaulon cylindratum

A.Oliveira & C.P.Bove

##### Materials

- **Type status:** Other material. **Occurrence:** recordNumber: 267; recordedBy: C. P. Bove et al.; **Location:** country: Brazil; countryCode: BRA; stateProvince: Mato Grosso; locality: Água Boa-Cocalinhos road, 124 Km from Água Boa, Cristalino River; verbatimLatitude: 14°8'58.65"S; verbatimLongitude: 51°32'21.18"W; verbatimCoordinateSystem: degree minutes; **Event:** year: 1997; month: 10; day: 12; **Record Level:** institutionID: Museu Nacional Herbarium; institutionCode: R

#### Eriocaulon epapillosum

Ruhland

##### Materials

- **Type status:** Other material. **Occurrence:** recordNumber: 30127; recordedBy: R. L. Fróes; **Location:** country: Brazil; countryCode: BRA; stateProvince: Goiás; locality: Couto Magalhães, Campos Gerais, Araguaia River; verbatimLatitude: 8°16'59.72"S; verbatimLongitude: 49°14'49.53"W; verbatimCoordinateSystem: degree minutes; **Event:** year: 1953; month: 8; day: 5; **Record Level:** institutionID: Museu Nacional Herbarium; institutionCode: R

#### Eriocaulon alto-gibbosum

Ruhland

##### Materials

- **Type status:** Other material. **Occurrence:** recordNumber: 8571; recordedBy: G. Eiten & L.Eiten; **Location:** country: Brazil; countryCode: BRA; stateProvince: Mato Grosso; locality: Barra do Garça, ca. 265 Km along road NNE of village of Xavantina; verbatimLatitude: 12°51'00.0"S; verbatimLongitude: 51°45'00.0"W; verbatimCoordinateSystem: degree minutes; **Event:** year: 1968; month: 9; day: 5; **Record Level:** institutionID: Universidade de Brasília Herbarium; institutionCode: UB

#### Eriocaulon humboldtii

Kunth

##### Materials

- **Type status:** Other material. **Occurrence:** recordNumber: 56270; recordedBy: B. Maguire; **Location:** country: Brazil; countryCode: BRA; stateProvince: Mato Grosso; locality: 15-120 Km beyond Alto Araguaia, road to Cuiabá, Brasilia-Acre Highway; verbatimLatitude: 17°18'39.01"S; verbatimLongitude: 53°13'22.93"W; verbatimCoordinateSystem: degree minutes; **Event:** year: 1963; month: 8; day: 25; **Record Level:** institutionID: Universidade de Brasília Herbarium; institutionCode: UB

#### Eriocaulon setaceum

L.

##### Materials

- **Type status:** Other material. **Occurrence:** recordNumber: 1679; recordedBy: C. P. Bove et al.; **Location:** country: Brazil; countryCode: BRA; stateProvince: Tocantins; locality: BR 242, Formoso do Araguaia- São João do Javaés Highway; verbatimLatitude: 11°47'36"S; verbatimLongitude: 49°43'31"W; verbatimCoordinateSystem: degree minutes; **Event:** year: 2004; month: 6; day: 15; **Record Level:** institutionID: Museu Nacional Herbarium; institutionCode: R

#### Comanthera xeranthemoides

(Bong.) L. R. Parra & Giul.

##### Materials

- **Type status:** Other material. **Occurrence:** recordNumber: 657; recordedBy: C. P. Bove et al.; **Location:** country: Brazil; countryCode: BRA; stateProvince: Goiás; locality: Caiapônia-Piranhas road, 30 Km from Caiapônia, dam of Samambaia farm; verbatimLatitude: 16°42'27.83"S; verbatimLongitude: 51°40'50.90"W; verbatimCoordinateSystem: degree minutes; **Event:** year: 1999; month: 11; day: 18; **Record Level:** institutionID: Museu Nacional Herbarium; institutionCode: R

#### Paepalanthus viridis

Körn.

##### Materials

- **Type status:** Other material. **Occurrence:** recordNumber: 305; recordedBy: R. César; **Location:** country: Brazil; countryCode: BRA; stateProvince: Goiás; locality: Caiapônia, Bela Vista farm; verbatimLatitude: 16°57'15.57"S; verbatimLongitude: 51°48'34.36"W; verbatimCoordinateSystem: degree minutes; **Event:** year: 1995; month: 4; day: 30; **Record Level:** institutionID: Universidade Federal de Goiás Herbarium; institutionCode: UFG

#### Syngonanthus caulescens

(Poir.) Ruhland

##### Materials

- **Type status:** Other material. **Occurrence:** recordNumber: 166; recordedBy: C. P. Bove et al.; **Location:** country: Brazil; countryCode: BRA; stateProvince: Goiás; locality: Jussara-Aragarças road, Km 100; verbatimLatitude: 14°49'10"S; verbatimLongitude: 50°58'36"W; verbatimCoordinateSystem: degree minutes; **Event:** year: 1997; month: 6; day: 26; **Record Level:** institutionID: Museu Nacional Herbarium; institutionCode: R

#### Syngonanthus densiflorus

(Körn.) Ruhland

##### Materials

- **Type status:** Other material. **Occurrence:** recordNumber: 17022; recordedBy: H. S. Irwin; **Location:** country: Brazil; countryCode: BRA; stateProvince: Mato Grosso; locality: Barra do Garças, ca. 30 Km South of Xavantina, drainage of the upper Araguaia River; verbatimLatitude: 15°53'25.1"S; verbatimLongitude: 52°15'24.1"W; verbatimCoordinateSystem: degree minutes; **Event:** year: 1966; month: 6; day: 12; **Record Level:** institutionID: The New York Botanical Garden Herbarium; institutionCode: NY

#### Syngonanthus gracilis

(Bong.) Ruhland

##### Materials

- **Type status:** Other material. **Occurrence:** recordNumber: 304; recordedBy: R. César; **Location:** country: Brazil; countryCode: BRA; stateProvince: Goiás; locality: Caiapônia-Iporá; verbatimLatitude: 16°57'15.57"S; verbatimLongitude: 51°48'34.36"W; verbatimCoordinateSystem: degree minutes; **Event:** year: 1995; month: 4; day: 30; **Record Level:** institutionID: Museu Nacional Herbarium; institutionCode: R

#### Syngonanthus helminthorrihizus

(Mart.) Ruhland

##### Materials

- **Type status:** Other material. **Occurrence:** recordNumber: 659; recordedBy: C. P. Bove et al.; **Location:** country: Brazil; countryCode: BRA; stateProvince: Goiás; locality: 30 kmfrom Caiapônia, dam of Samambaia farm; verbatimLatitude: 16°42'27.83"S; verbatimLongitude: 51°40'50.90"W; verbatimCoordinateSystem: degree minutes; **Event:** year: 1999; month: 11; day: 18; **Record Level:** institutionID: Museu Nacional Herbarium; institutionCode: R

#### Syngonanthus humboldtii

(Kunth) Ruhland

##### Materials

- **Type status:** Other material. **Occurrence:** recordNumber: 1636; recordedBy: F.C.A. Oliveira; **Location:** country: Brazil; countryCode: BRA; stateProvince: Tocantins; locality: Sandolândia, Araguaia River basin, Javaés River; verbatimLatitude: 12°29'29.0"S; verbatimLongitude: 50°06'42.0"W; verbatimCoordinateSystem: degree minutes; **Event:** year: 2009; month: 7; day: 12; **Record Level:** institutionID: Universidade Estadual de Feira de Santana Herbarium; institutionCode: HUEFS

#### Syngonanthus nitens

(Bong.) Ruhland

##### Materials

- **Type status:** Other material. **Occurrence:** recordNumber: 266; recordedBy: D.M.S. Rocha; **Location:** country: Brazil; countryCode: BRA; stateProvince: Goiás; locality: Santa Rita do Araguaia, bridge over Babilônia River; verbatimLatitude: 17°19'30"S; verbatimLongitude: 53°12'18"W; verbatimCoordinateSystem: degree minutes; **Event:** year: 2000; month: 3; day: 29; **Record Level:** institutionID: Universidade de Brasília Herbarium; institutionCode: UB

#### Syngonanthus longipes

Gleason

##### Materials

- **Type status:** Other material. **Occurrence:** recordNumber: 35034; recordedBy: G. Hatschbach; **Location:** country: Brazil; countryCode: BRA; stateProvince: Mato Grosso; locality: Alto Araguaia, Sapo stream; verbatimLatitude: 17°18'64"S; verbatimLongitude: 53°13'08"W; verbatimCoordinateSystem: degree minutes; **Event:** year: 1974; month: 11; day: 21; **Record Level:** institutionID: Missouri Botanical Garden Herbarium; institutionCode: MO

#### Tonina fluviatilis

Aubl.

##### Materials

- **Type status:** Other material. **Occurrence:** recordNumber: 9122; recordedBy: T. Plowman et al.; **Location:** country: Brazil; countryCode: BRA; stateProvince: Pará; locality: Conceição do Araguaia, 2 Km West of town, along highway PA-287; verbatimLatitude: 8°15'00.0"S; verbatimLongitude: 49°18'00.0"W; verbatimCoordinateSystem: degree minutes; **Event:** year: 1980; month: 2; day: 24; **Record Level:** institutionID: Missouri Botanical Garden Herbarium; institutionCode: MO

#### Heliconia psittacorum

L.f.

##### Materials

- **Type status:** Other material. **Occurrence:** recordNumber: 1642; recordedBy: C. P. Bove et al.; **Location:** country: Brazil; countryCode: BRA; stateProvince: Goiás; locality: Jussara-Aragarças road, 100 Km from Jussara; verbatimLatitude: 15°53'39.52"S; verbatimLongitude: 51°26'14.56"W; verbatimCoordinateSystem: degree minutes; **Event:** year: 2006; month: 4; day: 12; **Record Level:** institutionID: Museu Nacional Herbarium; institutionCode: R

#### Elodea granatensis

Humb. & Bonpl.

##### Materials

- **Type status:** Other material. **Occurrence:** recordNumber: 224; recordedBy: C. P. Bove et al.; **Location:** country: Brazil; countryCode: BRA; stateProvince: Goiás; locality: Aruanã-Goiânia road; verbatimLatitude: 14°55'19.68"S; verbatimLongitude: 51°4'21.89"W; verbatimCoordinateSystem: degree minutes; **Event:** year: 1997; month: 5; day: 29; **Record Level:** institutionID: Museu Nacional Herbarium; institutionCode: R

#### Egeria heterostemon

S.Koehler & C.P.Bove

##### Materials

- **Type status:** Other material. **Occurrence:** recordNumber: 225; recordedBy: C. P. Bove et al.; **Location:** country: Brazil; countryCode: BRA; stateProvince: Goiás; locality: Aruanã-Goiânia road; verbatimLatitude: 14°55'19.68"S; verbatimLongitude: 51°4'21.89"W; verbatimCoordinateSystem: degree minutes; **Event:** year: 1997; month: 5; day: 29; **Record Level:** institutionID: Museu Nacional Herbarium; institutionCode: R

#### Najas affinis

Rendle

##### Materials

- **Type status:** Other material. **Occurrence:** recordNumber: 260; recordedBy: C. P. Bove et al.; **Location:** country: Brazil; countryCode: BRA; stateProvince: Goiás; locality: Água Boa-Cocalinho road; verbatimLatitude: 14°06'08"S; verbatimLongitude: 51°40'20"W; verbatimCoordinateSystem: degree minutes; **Event:** year: 1997; month: 10; day: 12; **Record Level:** institutionID: Museu Nacional Herbarium; institutionCode: R

#### Ottelia brasiliensis

(Planch.) Walp.

##### Materials

- **Type status:** Other material. **Occurrence:** recordNumber: 191; recordedBy: C. P. Bove et al.; **Location:** country: Brazil; countryCode: BRA; stateProvince: Goiás; locality: Jussara-Aragarças road; verbatimLatitude: 15°53'53"S; verbatimLongitude: 52°13'21"W; verbatimCoordinateSystem: degree minutes; **Event:** year: 1997; month: 6; day: 26; **Record Level:** institutionID: Museu Nacional Herbarium; institutionCode: R

#### Thalia geniculata

L.

##### Materials

- **Type status:** Other material. **Occurrence:** recordNumber: 603; recordedBy: C. P. Bove et al.; **Location:** country: Brazil; countryCode: BRA; stateProvince: Goiás; locality: Aruanã-Araguapaz road; verbatimLatitude: 14°58'00"S; verbatimLongitude: 50°52'00"W; verbatimCoordinateSystem: degree minutes; **Event:** year: 1999; month: 11; day: 15; **Record Level:** institutionID: Museu Nacional Herbarium; institutionCode: R

#### Mayaca fluviatilis

Aubl.

##### Materials

- **Type status:** Other material. **Occurrence:** recordNumber: 653; recordedBy: C. P. Bove et al.; **Location:** country: Brazil; countryCode: BRA; stateProvince: Goiás; locality: 30 Km from Caiapônia, Abóbora waterfall; verbatimLatitude: 16°42'27.83"S; verbatimLongitude: 51°40'50.90"W; verbatimCoordinateSystem: degree minutes; **Event:** year: 1999; month: 11; day: 18; **Record Level:** institutionID: Museu Nacional Herbarium; institutionCode: R

#### Mayaca longipes

Mart. ex Seub.

##### Materials

- **Type status:** Other material. **Occurrence:** recordNumber: 583; recordedBy: C. P. Bove et al.; **Location:** country: Brazil; countryCode: BRA; stateProvince: Goiás; locality: Jussara- Britânia road, 62.6 Km from Jussara, Pindaíba stream; verbatimLatitude: 15°33'10.31"S; verbatimLongitude: 51°14'24.05"W; verbatimCoordinateSystem: degree minutes; **Event:** year: 1999; month: 11; day: 14; **Record Level:** institutionID: Museu Nacional Herbarium; institutionCode: R

#### Mayaca madida

(Vell.) Stellfeld

##### Materials

- **Type status:** Other material. **Occurrence:** recordNumber: 248; recordedBy: C. P. Bove et al.; **Location:** country: Brazil; countryCode: BRA; stateProvince: Mato Grosso; locality: BR-158, 5 Km from Água Boa; verbatimLatitude: 14°05'38"S; verbatimLongitude: 52°09'53"W; verbatimCoordinateSystem: degree minutes; **Event:** year: 1997; month: 10; day: 10; **Record Level:** institutionID: Museu Nacional Herbarium; institutionCode: R

#### Bletia catenulata

Ruiz & Pav.

##### Materials

- **Type status:** Other material. **Occurrence:** recordNumber: s.n.; recordedBy: D. M. S. Rocha; **Location:** country: Brazil; countryCode: BRA; stateProvince: Goiás; locality: Santa Rita do Araguaia, Babilônia River, next to the bridge; verbatimLatitude: 17°19'33"S; verbatimLongitude: 53°12'11"W; verbatimCoordinateSystem: degree minutes; **Event:** year: 2000; month: 3; day: 22; **Record Level:** institutionID: Universidade de Brasília Herbarium; institutionCode: UB (15590)

#### Epidendrum densiflorum

Hook.

##### Materials

- **Type status:** Other material. **Occurrence:** recordNumber: 8857; recordedBy: T. Plowman et al.; **Location:** country: Brazil; countryCode: BRA; stateProvince: Pará; locality: Santana do Araguaia, Redenção-Barreiras dos Campos road, PA-150, 100 Km South of Redenção; verbatimLatitude: 8°45'00.0"S; verbatimLongitude: 50°25'12.0"W; verbatimCoordinateSystem: degree minutes; **Event:** year: 1980; month: 2; day: 18; **Record Level:** institutionID: Intituto Nacional de Pesquisas da Amazonia Herbarium; institutionCode: INPA

#### Habenaria macilenta

(Lindl. ex Benth.) Rchb.f.

##### Materials

- **Type status:** Other material. **Occurrence:** recordNumber: s.n.; recordedBy: R. S. Oliveira; **Location:** country: Brazil; countryCode: BRA; stateProvince: Goiás; locality: São Miguel do Araguaia- Luis Alves Tocantins road, ca. 33 Km from São Miguel do Araguaia; verbatimLatitude: 13°17'15.45"S; verbatimLongitude: 50°27'54.15"W; verbatimCoordinateSystem: degree minutes; **Event:** year: 1997; month: 2; day: 15; **Record Level:** institutionID: Universidade de Brasília Herbarium; institutionCode: (UB 19881)

#### Habenaria orchiocalcar

Hoehne

##### Materials

- **Type status:** Other material. **Occurrence:** recordNumber: s.n.; recordedBy: R. S. Oliveira; **Location:** country: Brazil; countryCode: BRA; stateProvince: Goiás; locality: São Miguel do Araguaia- Luis Alves Tocantins road, ca. 33 Km from São Miguel do Araguaia; verbatimLatitude: 13°17'15.45"S; verbatimLongitude: 50°27'54.15"W; verbatimCoordinateSystem: degree minutes; **Event:** year: 1997; month: 2; day: 15; **Record Level:** institutionID: Universidade de Brasília Herbarium; institutionCode: (UB 19882)

#### Habenaria spathulifera

Cogn.

##### Materials

- **Type status:** Other material. **Occurrence:** recordNumber: 17084; recordedBy: H. S. Irwin; **Location:** country: Brazil; countryCode: BRA; stateProvince: Mato Grosso; locality: Drainage of the upper Araguaia River; verbatimLatitude: 17°18'56.81"S; verbatimLongitude: 53°13'6.96"W; verbatimCoordinateSystem: degree minutes; **Event:** year: 1966; month: 6; day: 13; **Record Level:** institutionID: Instituto de Botânica Herbarium; institutionCode: SP

#### Acroceras fluminense

(Hack.) Zuloaga & Morrone

##### Materials

- **Type status:** Other material. **Occurrence:** recordNumber: 8647; recordedBy: T. Plowman et al.; **Location:** country: Brazil; countryCode: BRA; stateProvince: Pará; locality: Conceicão do Araguaia, West of Redencão near São João stream; verbatimLatitude: 8°3'S; verbatimLongitude: 50°10'W; verbatimCoordinateSystem: degree minutes; **Event:** year: 1980; month: 2; day: 10; **Record Level:** institutionID: Missouri Botanical Garden Herbarium; institutionCode: MO

#### Andropogon lateralis

Nees

##### Materials

- **Type status:** Other material. **Occurrence:** recordNumber: 657; recordedBy: A. Zanin; **Location:** country: Brazil; countryCode: BRA; stateProvince: Mato Grosso; locality: BR 364, ca. 90 Km of border of Goiás/Mato Grosso, road to Cuiabá; verbatimLatitude: 16°49'51.82"S; verbatimLongitude: 53°59'59.29"W; verbatimCoordinateSystem: degree minutes; **Event:** year: 1997; month: 11; day: 26; **Record Level:** institutionID: Universidade de Santa Catarina Herbarium; institutionCode: FLOR

#### Andropogon leucostachyus

Kunth

##### Materials

- **Type status:** Other material. **Occurrence:** recordNumber: 14556; recordedBy: V. C. Souza et al.; **Location:** country: Brazil; countryCode: BRA; stateProvince: Mato Grosso; locality: road to Mata do Coco, 14 Km North from Luciara; verbatimLatitude: 11°04'50.9"S; verbatimLongitude: 50°26'04.2"W; verbatimCoordinateSystem: degree minutes; **Event:** year: 1997; month: 3; day: 19; **Record Level:** institutionID: Universidade de São Paulo Herbarium; institutionCode: ESA

#### Digitaria ciliaris

(Retz.) Koeler

##### Materials

- **Type status:** Other material. **Occurrence:** recordNumber: s.n.; recordedBy: H. D. Ferreira; **Location:** country: Brazil; countryCode: BRA; stateProvince: Goiás; locality: Luiz Alves; verbatimLatitude: 26°42'37"S; verbatimLongitude: 48°54'42.52"W; verbatimCoordinateSystem: degree minutes; **Record Level:** institutionID: Universidade Federal de Goiás Herbarium; institutionCode: (UFG 13986)

#### Digitaria filiformis

(L.) Kaler

##### Materials

- **Type status:** Other material. **Occurrence:** recordNumber: 499; recordedBy: C. P. Bove et al.; **Location:** country: Brazil; countryCode: BRA; stateProvince: Goiás; locality: Jussara-Aragarças road; verbatimLatitude: 15°53'53"S; verbatimLongitude: 52°13'21"W; verbatimCoordinateSystem: degree minutes; **Event:** year: 1999; month: 11; day: 11; **Record Level:** institutionID: Museu Nacional Herbarium; institutionCode: R

#### Echinochloa colona

(L.) Link

##### Materials

- **Type status:** Other material. **Occurrence:** recordNumber: 1670; recordedBy: C. P. Bove et al.; **Location:** country: Brazil; countryCode: BRA; stateProvince: Tocantins; locality: Formoso do Araguaia-São João do Javaés road; verbatimLatitude: 11°47'36"S; verbatimLongitude: 49°43'31"W; verbatimCoordinateSystem: degree minutes; **Event:** year: 2004; month: 6; day: 15; **Record Level:** institutionID: Museu Nacional Herbarium; institutionCode: R

#### Echinochloa crus-galli

(L.) P.Beauv.

##### Materials

- **Type status:** Other material. **Occurrence:** recordNumber: 14471; recordedBy: V. C. Souza et al.; **Location:** country: Brazil; countryCode: BRA; stateProvince: Mato Grosso; locality: São Félix do Araguaia -Alto da Boa Vista road, ca. 9 Km from São Félix do Araguaia; verbatimLatitude: 11°37'58.0"S; verbatimLongitude: 50°46'34.0"W; verbatimCoordinateSystem: degree minutes; **Event:** year: 1997; month: 10; day: 26; **Record Level:** institutionID: Universidade de São Paulo Herbarium; institutionCode: ESA

#### Eleusine indica

(L.) Gaertn.

##### Materials

- **Type status:** Other material. **Occurrence:** recordNumber: 17677; recordedBy: H. S. Irwin et al.; **Location:** country: Brazil; countryCode: BRA; stateProvince: Mato Grosso; locality: Piranhas, Drainage of the Upper Araguaia River; verbatimLatitude: 16°31'48.0"S; verbatimLongitude: 51°51'00.0"W; verbatimCoordinateSystem: degree minutes; **Event:** year: 1966; month: 6; day: 23; **Record Level:** institutionID: Missouri Botanical Garden Herbarium; institutionCode: MO

#### Eragrostis japonica

(Thunb.) Trin.

##### Materials

- **Type status:** Other material. **Occurrence:** recordNumber: s. n.; recordedBy: H. D. Ferreira; **Location:** country: Brazil; countryCode: BRA; stateProvince: Goiás; locality: Luiz Alves; verbatimLatitude: 26°42'37"S; verbatimLongitude: 48°54'42.52"W; verbatimCoordinateSystem: degree minutes; **Record Level:** institutionID: Universidade Federal de Goiás Herbarium; institutionCode: (UFG 13985)

#### Eragrostis rufescens

Schult.

##### Materials

- **Type status:** Other material. **Occurrence:** recordNumber: 134; recordedBy: H. Jancoski; **Location:** country: Brazil; countryCode: BRA; stateProvince: Mato Grosso; locality: Novo Santo Antônio, State Park of Araguaia, Silvana farm; verbatimLatitude: 12°17'26"S; verbatimLongitude: 50°58'05"W; verbatimCoordinateSystem: degree minutes; **Event:** year: 2005; month: 6; day: 5; **Record Level:** institutionID: Universidade Federal do Mato Grosso do Sul; institutionCode: CGMS

#### Eriochrysis cayennensis

P. Beauv.

##### Materials

- **Type status:** Other material. **Occurrence:** recordNumber: 278; recordedBy: D. M. S. Rocha; **Location:** country: Brazil; countryCode: BRA; stateProvince: Goiás; locality: Santa Rita do Araguaia, bridge over Babilônia River; verbatimLatitude: 17°19'33"S; verbatimLongitude: 53°12'11"W; verbatimCoordinateSystem: degree minutes; **Event:** year: 2000; month: 3; day: 29; **Record Level:** institutionID: Universidade de Brasília Herbarium; institutionCode: UB

#### Hymenachne amplexicaulis

(Rudge) Nees

##### Materials

- **Type status:** Other material. **Occurrence:** recordNumber: 629; recordedBy: C. P. Bove et al.; **Location:** country: Brazil; countryCode: BRA; stateProvince: Goiás; locality: GO-334, road to Peixe; verbatimLatitude: 14°52'27"S; verbatimLongitude: 50°30'16"W; verbatimCoordinateSystem: degree minutes; **Event:** year: 1999; month: 11; day: 16; **Record Level:** institutionID: Museu Nacional Herbarium; institutionCode: R

#### Mesosetum cf. loliiforme

(Steud.) Hitchc.

##### Materials

- **Type status:** Other material. **Occurrence:** recordNumber: 14803; recordedBy: V. C. Souza et al.; **Location:** country: Brazil; countryCode: BRA; stateProvince: Mato Grosso; locality: São Félix do Araguaia, Pontinópolis town-Serra dos Magalhães road; verbatimLatitude: 11°20'02.0"S; verbatimLongitude: 51°07'48.0"W; verbatimCoordinateSystem: degree minutes; **Event:** year: 1997; month: 3; day: 21; **Record Level:** institutionID: Universidade de São Paulo Herbarium; institutionCode: ESA

#### Ichnanthus procurrens

(Nees ex Trin.) Swallen

##### Materials

- **Type status:** Other material. **Occurrence:** recordNumber: 1664; recordedBy: C. P. Bove et al.; **Location:** country: Brazil; countryCode: BRA; stateProvince: Goiás; locality: GO-184, South of Nova Crixás; verbatimLatitude: 18°01'21"S; verbatimLongitude: 51°49'37"W; verbatimCoordinateSystem: degree minutes; **Event:** year: 2006; month: 4; day: 14; **Record Level:** institutionID: Museu Nacional Herbarium; institutionCode: R

#### Isachne polygonoides

(Lam.) Döll

##### Materials

- **Type status:** Other material. **Occurrence:** recordNumber: 651; recordedBy: C. P. Bove et al.; **Location:** country: Brazil; countryCode: BRA; stateProvince: Goiás; locality: 30 Km from Caiapônia; verbatimLatitude: 16°57'64"S; verbatimLongitude: 51°48'27"W; verbatimCoordinateSystem: degree minutes; **Event:** year: 1999; month: 11; day: 18; **Record Level:** institutionID: Museu Nacional Herbarium; institutionCode: R

#### Luziola peruviana

Juss ex J.F. Gmel.

##### Materials

- **Type status:** Other material. **Occurrence:** recordNumber: 247; recordedBy: C. P. Bove et al.; **Location:** country: Brazil; countryCode: BRA; stateProvince: Mato Grosso; locality: Barra do Garças-Água Boa road; verbatimLatitude: 15°53'36.60"S; verbatimLongitude: 52°15'47.19"W; verbatimCoordinateSystem: degree minutes; **Event:** year: 1997; month: 10; day: 10; **Record Level:** institutionID: Museu Nacional Herbarium; institutionCode: R

#### Panicum cyanescens

Nees ex Trin.

##### Materials

- **Type status:** Other material. **Occurrence:** recordNumber: 9103; recordedBy: T. Plowman et al.; **Location:** country: Brazil; countryCode: BRA; stateProvince: Pará; locality: Conceição do Araguaia, 2 Km West of town, along highway PA-287; verbatimLatitude: 8°15'00.0"S; verbatimLongitude: 49°18'00.0"W; verbatimCoordinateSystem: degree minutes; **Event:** year: 1980; month: 2; day: 24; **Record Level:** institutionID: Missouri Botanical Garden Herbarium; institutionCode: MO

#### Panicum discrepans

Döll

##### Materials

- **Type status:** Other material. **Occurrence:** recordNumber: 587A; recordedBy: C. P. Bove et al.; **Location:** country: Brazil; countryCode: BRA; stateProvince: Goiás; locality: Aruanã-Araguapaz road; verbatimLatitude: 14°58'00"S; verbatimLongitude: 50°52'00"W; verbatimCoordinateSystem: degree minutes; **Event:** year: 1999; month: 11; day: 15; **Record Level:** institutionID: Museu Nacional Herbarium; institutionCode: R

#### Panicum latissimum

J.G.Mikan ex Trin.

##### Materials

- **Type status:** Other material. **Occurrence:** recordNumber: 9107; recordedBy: T. Plowman et al.; **Location:** country: Brazil; countryCode: BRA; stateProvince: Pará; locality: Conceição do Araguaia, 2 Km West of town, along highway PA-287; verbatimLatitude: 8°15'00.0"S; verbatimLongitude: 49°18'00.0"W; verbatimCoordinateSystem: degree minutes; **Event:** year: 1980; month: 2; day: 24; **Record Level:** institutionID: Intituto Nacional de Pesquisas da Amazonia Herbarium; institutionCode: INPA

#### Panicum parvifolium

Lam.

##### Materials

- **Type status:** Other material. **Occurrence:** recordNumber: 557; recordedBy: C. P. Bove et al.; **Location:** country: Brazil; countryCode: BRA; stateProvince: Goiás; locality: Jussara-Britânia road; verbatimLatitude: 15°52'29.02"S; verbatimLongitude: 50°51'50.59"W; verbatimCoordinateSystem: degree minutes; **Event:** year: 1999; month: 11; day: 11; **Record Level:** institutionID: Museu Nacional Herbarium; institutionCode: R

#### Panicum repens

Berg.

##### Materials

- **Type status:** Other material. **Occurrence:** recordNumber: 502; recordedBy: C. P. Bove et al.; **Location:** country: Brazil; countryCode: BRA; stateProvince: Goiás; locality: Jussara-Aragarças road; verbatimLatitude: 15°53'53"S; verbatimLongitude: 52°13'21"W; verbatimCoordinateSystem: degree minutes; **Event:** year: 1999; month: 11; day: 11; **Record Level:** institutionID: Museu Nacional Herbarium; institutionCode: R

#### Panicum schwackeanum

Mez.

##### Materials

- **Type status:** Other material. **Occurrence:** recordNumber: 1663; recordedBy: C. P. Bove et al.; **Location:** country: Brazil; countryCode: BRA; stateProvince: Goiás; locality: GO-184, South of Nova Crixás; verbatimLatitude: 18°01'21"S; verbatimLongitude: 51°49'37"W; verbatimCoordinateSystem: degree minutes; **Event:** year: 2006; month: 4; day: 11; **Record Level:** institutionID: Museu Nacional Herbarium; institutionCode: R

#### Panicum stenodes

Griseb.

##### Materials

- **Type status:** Other material. **Occurrence:** recordNumber: 17016; recordedBy: H.S. Irwin et al.; **Location:** country: Brazil; countryCode: BRA; stateProvince: Mato Grosso; locality: ca. 30 Km South of Xavantina, drainage of the Upper Araguaia River; verbatimLatitude: 14°55'12.0"S; verbatimLongitude: 52°19'12.0"W; verbatimCoordinateSystem: degree minutes; **Event:** year: 1966; month: 6; day: 12; **Record Level:** institutionID: Missouri Botanical Garden Herbarium; institutionCode: MO

#### Panicum trichoides

Sw.

##### Materials

- **Type status:** Other material. **Occurrence:** recordNumber: 14466; recordedBy: V. C. Souza et al.; **Location:** country: Brazil; countryCode: BRA; stateProvince: Mato Grosso; locality: São Félix do Araguaia-Alto da Boa Vista road, ca. 9 Km from São Félix do Araguaia; verbatimLatitude: 11°22'32.9"S; verbatimLongitude: 50°27'48.2"W; verbatimCoordinateSystem: degree minutes; **Event:** year: 1997; month: 3; day: 18; **Record Level:** institutionID: Universidade de São Paulo Herbarium; institutionCode: ESA

#### Paspalum cinerascens

(Döll) A.G.Burm. & M.Bastos

##### Materials

- **Type status:** Other material. **Occurrence:** recordNumber: 9098; recordedBy: T. Plowman et al.; **Location:** country: Brazil; countryCode: BRA; stateProvince: Pará; locality: Conceição do Araguaia, 2 Km West of town, along highway PA-287; verbatimLatitude: 8°15'00.0"S; verbatimLongitude: 49°18'00.0"W; verbatimCoordinateSystem: degree minutes; **Event:** year: 1980; month: 2; day: 24; **Record Level:** institutionID: Intituto Nacional de Pesquisas da Amazonia Herbarium; institutionCode: INPA

#### Paspalum convexum

Humb. & Bonpl. ex Flüggé

##### Materials

- **Type status:** Other material. **Occurrence:** recordNumber: 17676; recordedBy: H. S. Irwin et al.; **Location:** country: Brazil; countryCode: BRA; stateProvince: Goiás; locality: Piranhas, drainage of the Upper Araguaia River; verbatimLatitude: 16°31'48.0"S; verbatimLongitude: 51°51'00.0"W; verbatimCoordinateSystem: degree minutes; **Event:** year: 1966; month: 6; day: 23; **Record Level:** institutionID: Missouri Botanical Garden Herbarium; institutionCode: MO

#### Paspalum gardnerianum

Nees

##### Materials

- **Type status:** Other material. **Occurrence:** recordNumber: 14805; recordedBy: V. C. Souza et al.; **Location:** country: Brazil; countryCode: BRA; stateProvince: Mato Grosso; locality: Pontinópolis town-Serra dos Magalhães, road; verbatimLatitude: 11°33'39.4"S; verbatimLongitude: 51°13'00.0"W; verbatimCoordinateSystem: degree minutes; **Event:** year: 1997; month: 3; **Record Level:** institutionID: Universidade de São Paulo Herbarium; institutionCode: ESA

#### Paspalum glaucescens

Hack.

##### Materials

- **Type status:** Other material. **Occurrence:** recordNumber: 12055; recordedBy: A. Chase; **Location:** country: Brazil; countryCode: BRA; stateProvince: Goiás; locality: Santa Rita do Araguaia; verbatimLatitude: 17°19'33"S; verbatimLongitude: 53°12'11"W; verbatimCoordinateSystem: degree minutes; **Event:** year: 1930; month: 4; day: 15; **Record Level:** institutionID: Jardim Botânico do Rio de Janeiro Herbarium; institutionCode: RB

#### Paspalum maritimum

Trin.

##### Materials

- **Type status:** Other material. **Occurrence:** recordNumber: 9104; recordedBy: T. Plowman et al.; **Location:** country: Brazil; countryCode: BRA; stateProvince: Pará; locality: Conceição do Araguaia, 2 Km West of town, along highway PA-287; verbatimLatitude: 8°15'00.0"S; verbatimLongitude: 49°18'00.0"W; verbatimCoordinateSystem: degree minutes; **Event:** year: 1980; month: 2; day: 24; **Record Level:** institutionID: Missouri Botanical Garden Herbarium; institutionCode: MO

#### Paspalum marmoratum

Kuhlm.

##### Materials

- **Type status:** Other material. **Occurrence:** recordNumber: 11866; recordedBy: A. Chase; **Location:** country: Brazil; countryCode: BRA; stateProvince: Goiás; locality: Santa Rita do Araguaia; verbatimLatitude: 17°19'33"S; verbatimLongitude: 53°12'11"W; verbatimCoordinateSystem: degree minutes; **Event:** year: 1930; month: 4; day: 7; **Record Level:** institutionID: Museu Nacional Herbarium; institutionCode: R

#### Paspalum pictum

Eckm.

##### Materials

- **Type status:** Other material. **Occurrence:** recordNumber: 12045; recordedBy: A. Chase; **Location:** country: Brazil; countryCode: BRA; stateProvince: Goiás; locality: Santa Rita do Araguaia; verbatimLatitude: 17°19'33"S; verbatimLongitude: 53°12'11"W; verbatimCoordinateSystem: degree minutes; **Event:** year: 1930; month: 4; day: 15; **Record Level:** institutionID: Museu Nacional Herbarium; institutionCode: R

#### Paspalum repens

Berg.

##### Materials

- **Type status:** Other material. **Occurrence:** recordNumber: 611; recordedBy: C. P. Bove et al.; **Location:** country: Brazil; countryCode: BRA; stateProvince: Goiás; locality: Mozarlândia-Nova Crixás road; verbatimLatitude: 14°27'00"S; verbatimLongitude: 50°27'00"W; verbatimCoordinateSystem: degree minutes; **Event:** year: 1999; month: 11; day: 15; **Record Level:** institutionID: Museu Nacional Herbarium; institutionCode: R

#### Paspalum trichotomum

Hack.

##### Materials

- **Type status:** Other material. **Occurrence:** recordNumber: 1182; recordedBy: A. Chase; **Location:** country: Brazil; countryCode: BRA; stateProvince: Goiás; locality: Santa Rita do Araguaia, Congolha River; verbatimLatitude: 17°19'33"S; verbatimLongitude: 53°12'11"W; verbatimCoordinateSystem: degree minutes; **Event:** year: 1930; month: 4; day: 7; **Record Level:** institutionID: Jardim Botânico do Rio de Janeiro Herbarium; institutionCode: RB

#### Reimarochloa acuta

(Fluggé) Hitchc.

##### Materials

- **Type status:** Other material. **Occurrence:** recordNumber: 587b; recordedBy: C. P. Bove et al.; **Location:** country: Brazil; countryCode: BRA; stateProvince: Goiás; locality: Aruanã-Araguapaz road; verbatimLatitude: 14°58'00"S; verbatimLongitude: 50°52'00"W; verbatimCoordinateSystem: degree minutes; **Event:** year: 1999; month: 11; day: 15; **Record Level:** institutionID: Museu Nacional Herbarium; institutionCode: R

#### Sacciolepis vilvoides

(Trin.) Chase

##### Materials

- **Type status:** Other material. **Occurrence:** recordNumber: 140; recordedBy: H. Jancoski; **Location:** country: Brazil; countryCode: BRA; stateProvince: Mato Grosso; locality: Novo Santo Antônio, State Park of Araguaia. Silvana farm; verbatimLatitude: 12°17'26"S; verbatimLongitude: 50°58'05"W; verbatimCoordinateSystem: degree minutes; **Event:** year: 2005; month: 6; day: 5; **Record Level:** institutionID: Universidade Federal do Mato Grosso do Sul; institutionCode: CGMS

#### Setaria parviflora

(Poir.) M.Kerguelen

##### Materials

- **Type status:** Other material. **Occurrence:** recordNumber: 8768; recordedBy: T. Plowman et al.; **Location:** country: Brazil; countryCode: BRA; stateProvince: Pará; locality: Conceição do Araguaia, ca. 20 Km West of Redenção, near São João stream; verbatimLatitude: 8°03'00.0"S; verbatimLongitude: 50°10'00.0"W; verbatimCoordinateSystem: degree minutes; **Event:** year: 1980; month: 2; day: 12; **Record Level:** institutionID: Missouri Botanical Garden Herbarium; institutionCode: MO

#### Steinchisma decipiens

(Nees ex Trin.) W.V.Br.

##### Materials

- **Type status:** Other material. **Occurrence:** recordNumber: 516; recordedBy: C. P. Bove et al.; **Location:** country: Brazil; countryCode: BRA; stateProvince: Goiás; locality: Jussara-Aragarças road, ca. 100 Km West from Jussara; verbatimLatitude: 15°52'36"S; verbatimLongitude: 51°49'31"W; verbatimCoordinateSystem: degree minutes; **Event:** year: 1999; month: 11; day: 11; **Record Level:** institutionID: Museu Nacional Herbarium; institutionCode: R

#### Steinchisma laxum

(Sw.) Zuloaga

##### Materials

- **Type status:** Other material. **Occurrence:** recordNumber: 1648; recordedBy: C. P. Bove et al.; **Location:** country: Brazil; countryCode: BRA; stateProvince: Goiás; locality: Aruanã-Canga road; verbatimLatitude: 14°44'38.8"S; verbatimLongitude: 50°56'34.81"W; verbatimCoordinateSystem: degree minutes; **Event:** year: 2006; month: 4; day: 13; **Record Level:** institutionID: Museu Nacional Herbarium; institutionCode: R

#### Thrasya thrasyoides

(Trin.) Chase

##### Materials

- **Type status:** Other material. **Occurrence:** recordNumber: 14695; recordedBy: V. C. Souza et al.; **Location:** country: Brazil; countryCode: BRA; stateProvince: Mato Grosso; locality: São Félix do Araguaia, ca. 31 Km WNW of São Félix do Araguaia, road to Luciara; verbatimLatitude: 11°35'19"S; verbatimLongitude: 50°54'26"W; verbatimCoordinateSystem: degree minutes; **Event:** year: 1997; month: 3; day: 20; **Record Level:** institutionID: Universidade de São Paulo Herbarium; institutionCode: ESA

#### Eichhornia azurea

(Sw.) Kunth

##### Materials

- **Type status:** Other material. **Occurrence:** recordNumber: 1799; recordedBy: C. P. Bove et al.; **Location:** country: Brazil; countryCode: BRA; stateProvince: Tocantins; locality: Alvorada-Peixe road; verbatimLatitude: 12°29'46"S; verbatimLongitude: 49°00'51"W; verbatimCoordinateSystem: degree minutes; **Event:** year: 2006; month: 4; day: 16; **Record Level:** institutionID: Museu Nacional Herbarium; institutionCode: R

#### Eichhornia crassipes

(Mart.) Solms

##### Materials

- **Type status:** Other material. **Occurrence:** recordNumber: 245; recordedBy: C. P. Bove et al.; **Location:** country: Brazil; countryCode: BRA; stateProvince: Goiás; locality: Jussara-Aragarças road; verbatimLatitude: 15°53'53"S; verbatimLongitude: 52°13'21"W; verbatimCoordinateSystem: degree minutes; **Event:** year: 1997; month: 10; day: 9; **Record Level:** institutionID: Museu Nacional Herbarium; institutionCode: R

#### Eichhornia diversifolia

(Vahl) Urb.

##### Materials

- **Type status:** Other material. **Occurrence:** recordNumber: 1692; recordedBy: C. P. Bove et al.; **Location:** country: Brazil; countryCode: BRA; stateProvince: Tocantins; locality: Alvorada-Peixe road; verbatimLatitude: 12°29'46"S; verbatimLongitude: 40°00'50"W; verbatimCoordinateSystem: degree minutes; **Event:** year: 2006; month: 4; day: 16; **Record Level:** institutionID: Museu Nacional Herbarium; institutionCode: R

#### Pontederia parviflora

Alexander

##### Materials

- **Type status:** Other material. **Occurrence:** recordNumber: 1688; recordedBy: C. P. Bove et al.; **Location:** country: Brazil; countryCode: BRA; stateProvince: Tocantins; locality: Alvorada-Peixe road; verbatimLatitude: 12°29'46.3"S; verbatimLongitude: 49°00'50"W; verbatimCoordinateSystem: degree minutes; **Event:** year: 2006; month: 4; day: 16; **Record Level:** institutionID: Museu Nacional Herbarium; institutionCode: R

#### Pontederia subovata

(Seub.) Lowden

##### Materials

- **Type status:** Other material. **Occurrence:** recordNumber: 1690; recordedBy: C. P. Bove et al.; **Location:** country: Brazil; countryCode: BRA; stateProvince: Tocantins; locality: Alvorada-Peixe road; verbatimLatitude: 12°29'46.3"S; verbatimLongitude: 49°00'50"W; verbatimCoordinateSystem: degree minutes; **Event:** year: 2006; month: 4; day: 16; **Record Level:** institutionID: Museu Nacional Herbarium; institutionCode: R

#### Cephalostemon gracilis

(Poepp. & Endl.) R.H.Schomb.

##### Materials

- **Type status:** Other material. **Occurrence:** recordNumber: 1323; recordedBy: C. P. Bove et al.; **Location:** country: Brazil; countryCode: BRA; stateProvince: Mato Grosso; locality: Alto Araguaia, Sapo stream; verbatimLatitude: 17°18'64"S; verbatimLongitude: 53°13'08"W; verbatimCoordinateSystem: degree minutes; **Event:** year: 2004; month: 1; day: 14; **Record Level:** institutionID: Museu Nacional Herbarium; institutionCode: R

#### Typha domingensis

Pers.

##### Materials

- **Type status:** Other material. **Occurrence:** recordNumber: 616; recordedBy: C. P. Bove et al.; **Location:** country: Brazil; countryCode: BRA; stateProvince: Goiás; locality: GO-334, road to Peixe, 8.8 Km from GO-164; verbatimLatitude: 14°52'27"S; verbatimLongitude: 50°30'16"W; verbatimCoordinateSystem: degree minutes; **Event:** year: 1999; month: 11; day: 16; **Record Level:** institutionID: Museu Nacional Herbarium; institutionCode: R

#### Abolboda pulchella

Bonpl.

##### Materials

- **Type status:** Other material. **Occurrence:** recordNumber: 14163; recordedBy: V. C. Souza; **Location:** country: Brazil; countryCode: BRA; stateProvince: Mato Grosso; locality: São Félix do Araguaia, ca. 13 Km, after entrance to Luciara.; verbatimLatitude: 11°34'21"S; verbatimLongitude: 50°59'13"W; verbatimCoordinateSystem: degree minutes; **Event:** year: 1997; month: 3; day: 15; **Record Level:** institutionID: Universidade Estadual de Campinas Herbarium; institutionCode: UEC

#### Xyris caroliniana

Walter

##### Materials

- **Type status:** Other material. **Occurrence:** recordNumber: 17473; recordedBy: H. S. Irwin; **Location:** country: Brazil; countryCode: BRA; stateProvince: Mato Grosso; locality: ca. 70 Km South of Xavantina, drainage of the Upper Araguaia River; verbatimLatitude: 15°16'12.0"S; verbatimLongitude: 52°15'00.0"W; verbatimCoordinateSystem: degree minutes; **Event:** year: 1966; month: 6; day: 19; **Record Level:** institutionID: The New York Botanical Garden Herbarium; institutionCode: NY

#### Xyris fragilis

Kral & Lor.B.Sm.

##### Materials

- **Type status:** Isotype. **Occurrence:** recordNumber: 8572; recordedBy: G. Eiten & L. T. Eiten; **Location:** country: Brazil; countryCode: BRA; stateProvince: Mato Grosso; locality: Barra do Garcas, 260 Km along new road NNE of village of Xavantina, Serra do Roncador; verbatimLatitude: 15°53'42"S; verbatimLongitude: 52°15'18"W; verbatimCoordinateSystem: degree minutes; **Event:** year: 1968; month: 9; day: 5; **Record Level:** institutionID: Missouri Botanical Garden Herbarium; institutionCode: MO

#### Xyris jupicai

Rich.

##### Materials

- **Type status:** Other material. **Occurrence:** recordNumber: 227; recordedBy: C. P. Bove et al.; **Location:** country: Brazil; countryCode: BRA; stateProvince: Goiás; locality: Jussara-Aragarças road, 100 Km from Jussara; verbatimLatitude: 15°53'19.94"S; verbatimLongitude: 51°18'19.02"W; verbatimCoordinateSystem: degree minutes; **Event:** year: 1997; month: 10; day: 9; **Record Level:** institutionID: Museu Nacional Herbarium; institutionCode: R

#### Xyris savanensis

Miq.

##### Materials

- **Type status:** Other material. **Occurrence:** recordNumber: 664; recordedBy: C. P. Bove et al.; **Location:** country: Brazil; countryCode: BRA; stateProvince: Goiás; locality: Caiapônia to Piranhas road, 30 Km from Caiapônia; verbatimLatitude: 16°47'12"S; verbatimLongitude: 51°43'16"W; verbatimCoordinateSystem: degree minutes; **Event:** year: 1999; month: 11; day: 18; **Record Level:** institutionID: Museu Nacional Herbarium; institutionCode: R

#### Xyris tenella

Kunth

##### Materials

- **Type status:** Other material. **Occurrence:** recordNumber: 6237a; recordedBy: J. A. Rizzo & A. Barbosa; **Location:** country: Brazil; countryCode: BRA; stateProvince: Goiás; locality: 40 Km from Amorinópolis, Verde River, Serra dos Caiapós; verbatimLatitude: 16°37'16"S; verbatimLongitude: 51°5'41"W; verbatimCoordinateSystem: degree minutes; **Event:** year: 1971; month: 4; day: 17; **Record Level:** institutionID: Universidade Federal de Goiás Herbarium; institutionCode: UFG

#### Xyris tortula

Mart.

##### Materials

- **Type status:** Other material. **Occurrence:** recordNumber: 34211; recordedBy: G. Hatschbach; **Location:** country: Brazil; countryCode: BRA; stateProvince: Mato Grosso; locality: Alto Araguaia, Rancho stream; verbatimLatitude: 17°14'55"S; verbatimLongitude: 53°15'00"W; verbatimCoordinateSystem: degree minutes; **Event:** year: 1974; month: 2; day: 14; **Record Level:** institutionID: Universidade Estadual de Campinas Herbarium; institutionCode: UEC

#### Hedychium coronarium

J.König

##### Materials

- **Type status:** Other material. **Occurrence:** recordNumber: 676; recordedBy: C. P. Bove et al.; **Location:** country: Brazil; countryCode: BRA; stateProvince: Goiás; locality: Iporá-São Luiz dos Montes Belos road, GO-060, 45.8 Km from Iporá; verbatimLatitude: 16°41'51"S; verbatimLongitude: 50°45'10"W; verbatimCoordinateSystem: degree minutes; **Event:** year: 1999; month: 11; day: 19; **Record Level:** institutionID: Museu Nacional Herbarium; institutionCode: R

## Discussion

**Figure 6.**
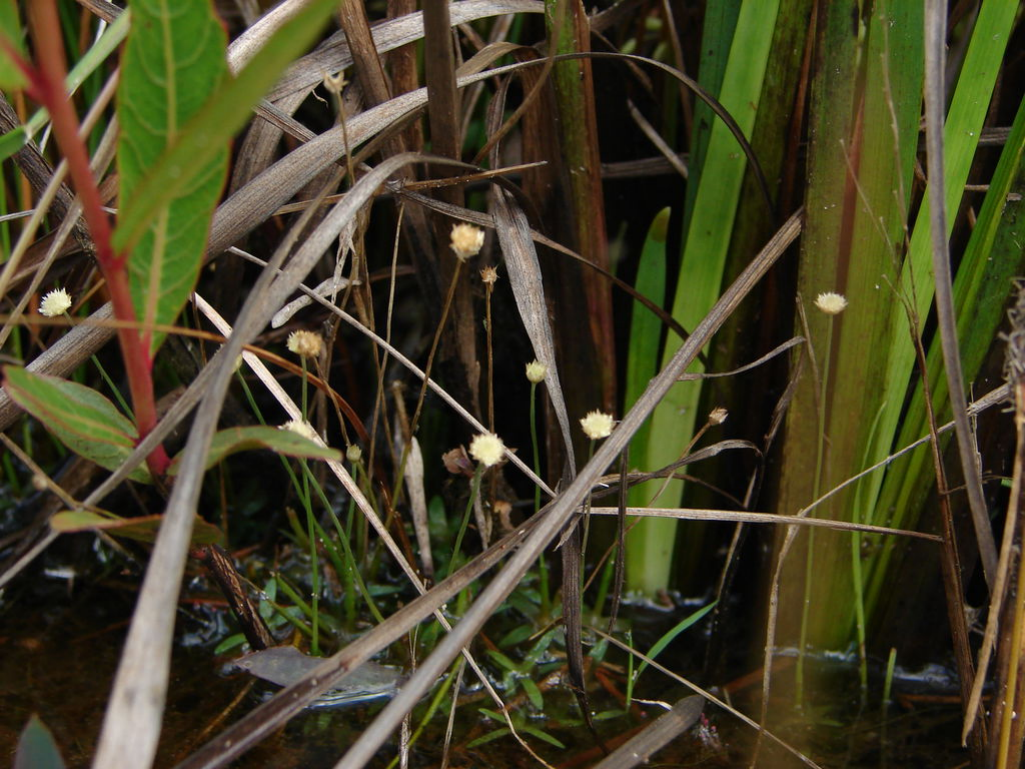
*Syngonanthus hirtellus* Ruhland habit.

We report 162 species of aquatic and marshy monocotyledons, distributed in 20 families and 50 genera (Table 1). Cyperaceae (51 spp.) (Fig. 2b), Poaceae (39 spp.) (Figs 2a, 3) and Eriocaulaceae (16 spp.) (Figs 4, 5, 6) are the most common families of this class. Among the monocots, the first two families are the most diverse in both aquatic and terrestrial ecosystems in Brazil (e.g. Moura-Júnior et al. 2013, Souza et al. 2009, Wanderley et al. 2011). Nonetheless, Eriocaulaceae has its greatest diversity in the Cerrado (e.g. IBGE 2004, Munhoz and Felfili 2006). Regarding life form, most plants are helophytes (98 spp.; 60.5%) or emergents (45 spp.; 27.8%); nine species floating (5.5%), six species emergent and/or submerged (3.7%), and four species are exclusively submerged (2.5%) (Table 1). The life form categories represent different degrees of adaptation to aquatic life and are widely convergent among aquatic angiosperms (Philbrick and Les 1996); so the high percentage of helophytes and emergent forms are expected for temporarily flooded swamps, pools and their margins.

In terms of floristics, 101 species are native to tropical and/or subtropical America, 40 are widely distributed and twenty are endemic to Brazil. Ninety-three species are new occurrences to the Araguaia River basin (Table 1). Floristic analysis generally emphasizes arboreal and shrub species. Among the studies that includes the herbaceous species; the aquatic taxa are rarely included or, at least, underestimated. This is reflected in the scarcity of aquatic vouchers in collections (Paz and Bove 2007). The few exceptions are herbaria associated with institutions having aquatic plant specialists. The checklist of the Emas National Park (Batalha and Martins 2002), cites 601 vascular species, nonetheless no strict aquatic taxa are included but only some helophytes as *Commelina obliqua* Vahl, *Rhynchospora rugosa* (Vahl) Gale and *Ichnanthus procurrens* (Nees ex Trin.) Swallen. Even in checklists from broader regions in Cerrado such as Vão do Paranã (Silva et al. 2004), only 13 out of 1273 vascular species are common with the list we produce herein. Clearly there is a need for more field work specifically focused on aquatic environments. Also, the detailed search through the database SpeciesLink (2002) added three families (Arecaceae, Cannaceae and Orchidaceae) and 62 species to the previous list based on field efforts (Table 1), showing the importance of a database as a tool in analyzing floristic diversity. The Araguaia River basin is far richer in aquatic and marshy species than previously realized. In addition to the new species described for this region mentioned above, the description of another new species of *Eriocaulon* is in progress.

Three species are not cited to the Brazilian Cerrado in any publication: *Panicum latissimum* J.G.Mikan ex Trin., *Philodendron imbe* Schott, and *Rhynchospora aff. unisetosa* T. Koyama. *Panicum latissimum* and *Philodendron imbe* are endemic from Brazil and *Rhynchospora unisetosa* is not reported to occur in Brazil (Alves et al. 2010) but it is found in Colombia and Venezuela; neighboring countries of Brazil (World Checklist of selected plant families 2013). The specimen identified as affinis *R. unisetosa* could also be another new species to the Araguaia region that needs attention from taxonomists. These species were, probably, overlooked and undercollected, highlighting the importance of field work by specialists, and points to the problem of relying on existing collections to determine real aquatic plant diversity. Incongruent data on geographic distribution and/or taxonomic identity were observed in some species; although *Syagrus petraea* (Mart.) Becc. and *Xyris caroliniana* Walter are recognized for the Cerrado and reported for the Araguaia basin using herbarium collections, these species are not mentioned in database of the Brazilian species list (Coelho et al. 2010, Wanderley et al. 2010 respectively). These apparent inconsistencies need the notice of the specialists to be resolved. We opted to keep these taxa in our survey in order to call attention to this matter and, moreover, we prefer to superestimate rather than subestimate the diversity. We consider that the consequences regarding conservation actions are worse given subestimates.

Since the 1970s, many projects have led to reduction in both diversity and area of wetlands. The construction of the Belém-Brasília highway and hydroelectric dams, as well as the expansion of agricultural and mining activities (Moss and Moss 2005), caused major impacts to wetlands in those areas. Notably, no species in this study is mentioned in the official list of Brazilian endangered species (Martinelli and Moraes 2013) probably as a result of insufficient data. *Burmannia flava* Mart. and *Paspalum cinerascens* (Döll) A.G.Burm. & M.Bastos are in redlists compiled by SpeciesLink (2002); *Eriocaulon araguaiense* and *E. cylindratum* are considered endangered by Oliveira and Bove (2015), both belonging to the ENB2 category (IUCN 2001).

## Figures and Tables

**Figure 2a.**
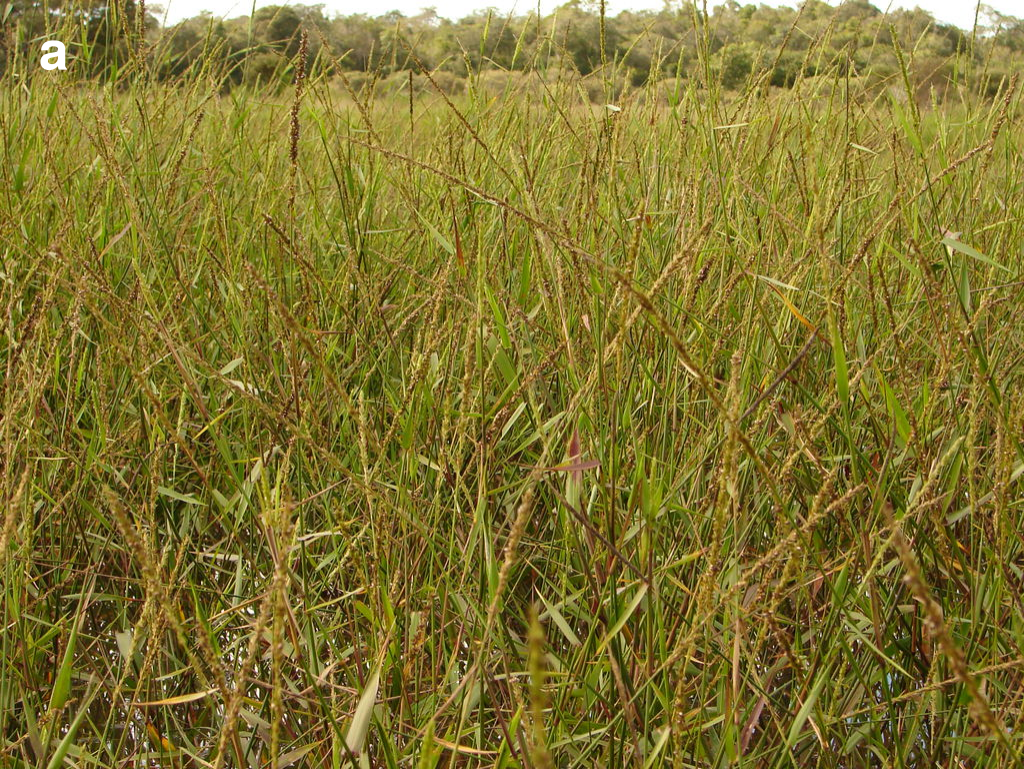
Temporay swamp dominated by *Hymenachne amplexicaulis* (Rudge) Nees. Goiás, Aruanã-Peixe road (14°44'34"S, 50°56' 34"W).

**Figure 2b.**
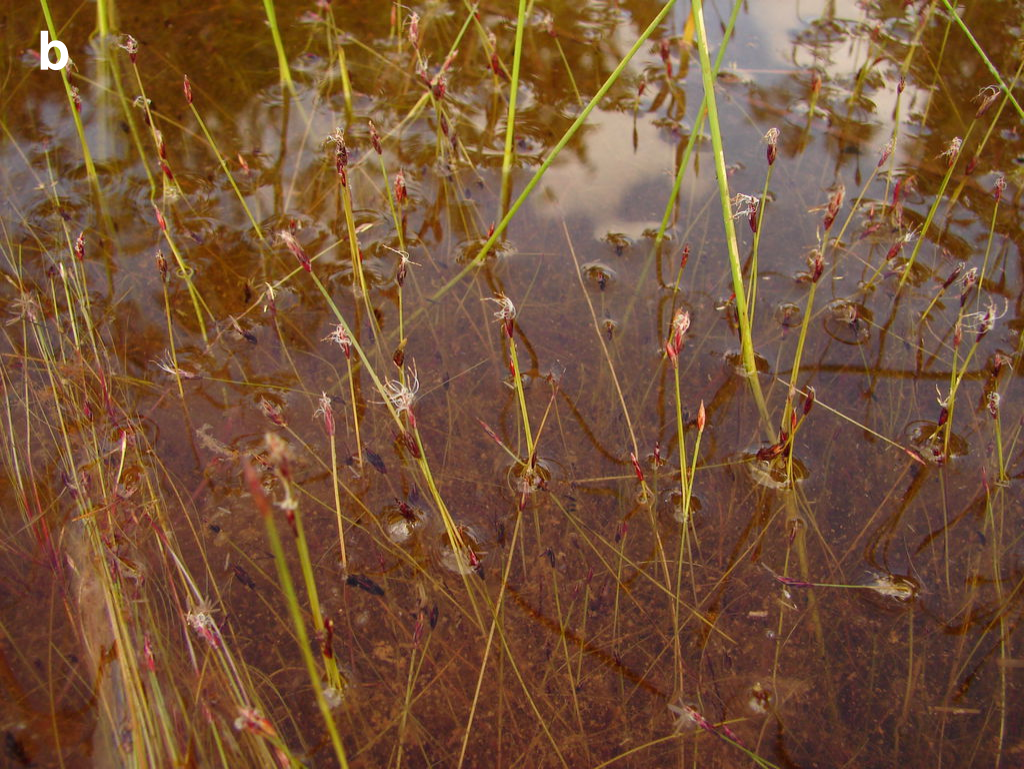
Temporay swamp dominated by *Eleocharis retroflexa* (Poir.) Urb. Goiás, Aruanã-Araguapaz road (14°49'10 "S, 50°58'36"W).

**Figure 2c.**
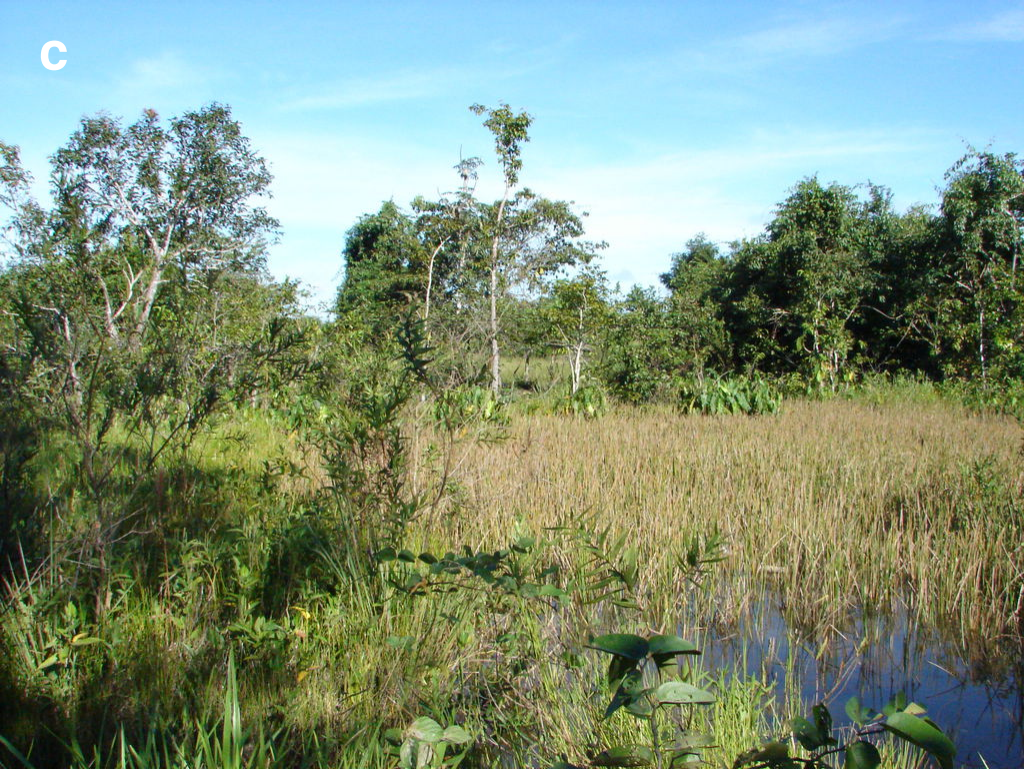
Temporay swamp dominated by Poaceae and Cyperaceae. Goiás, GO-184, 49,5km South of Nova Crixás.

**Figure 2d.**
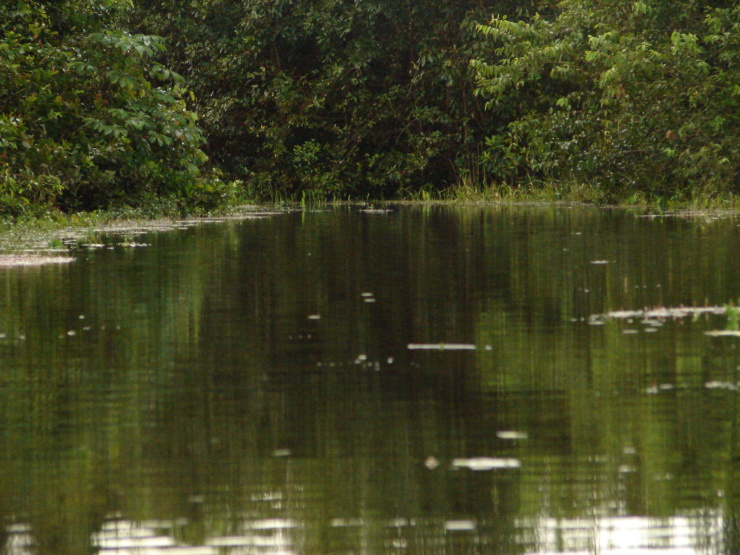
Permanent swamp with *Eriocaulon setaceum* L. Tocantins, BR-242, Formoso do Araguaia-São João do Javaés road.

**Figure 2e.**
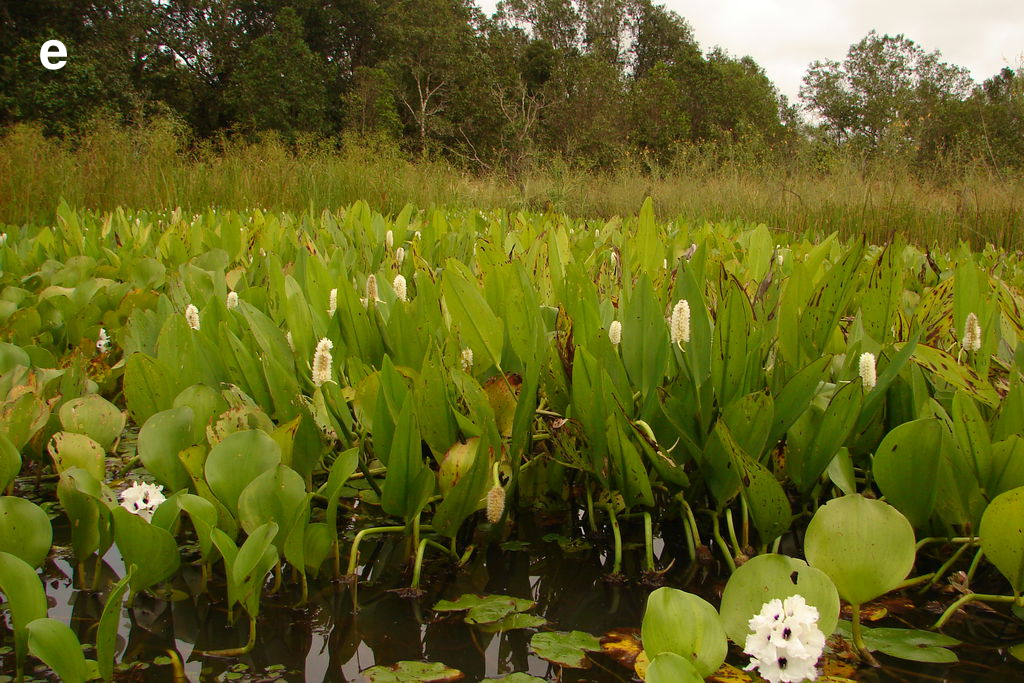
Temporay swamp dominated by Pontederiaceae. Tocantins, Alvorada-Peixe road (12°29'46"S, 49°00'51"W).

**Figure 2f.**
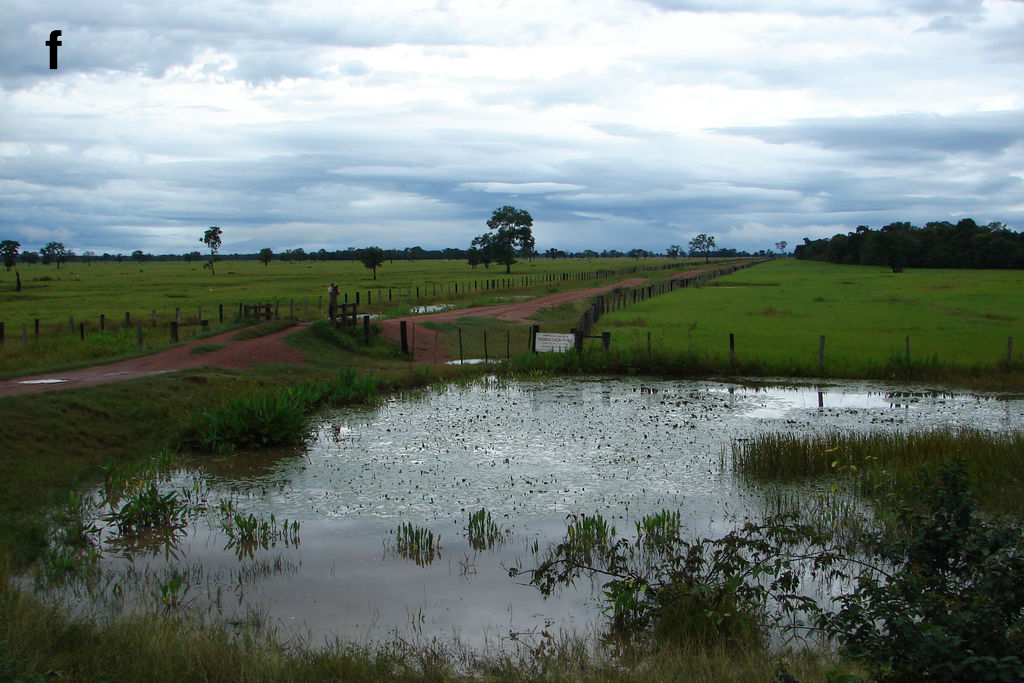
Temporary swamp with *Fimbristylis quinquangularis* (Vahl) Kunth. Tocantins, BR-242, Formoso do Araguaia-São João do Javaés road.

**Figure 3a.**
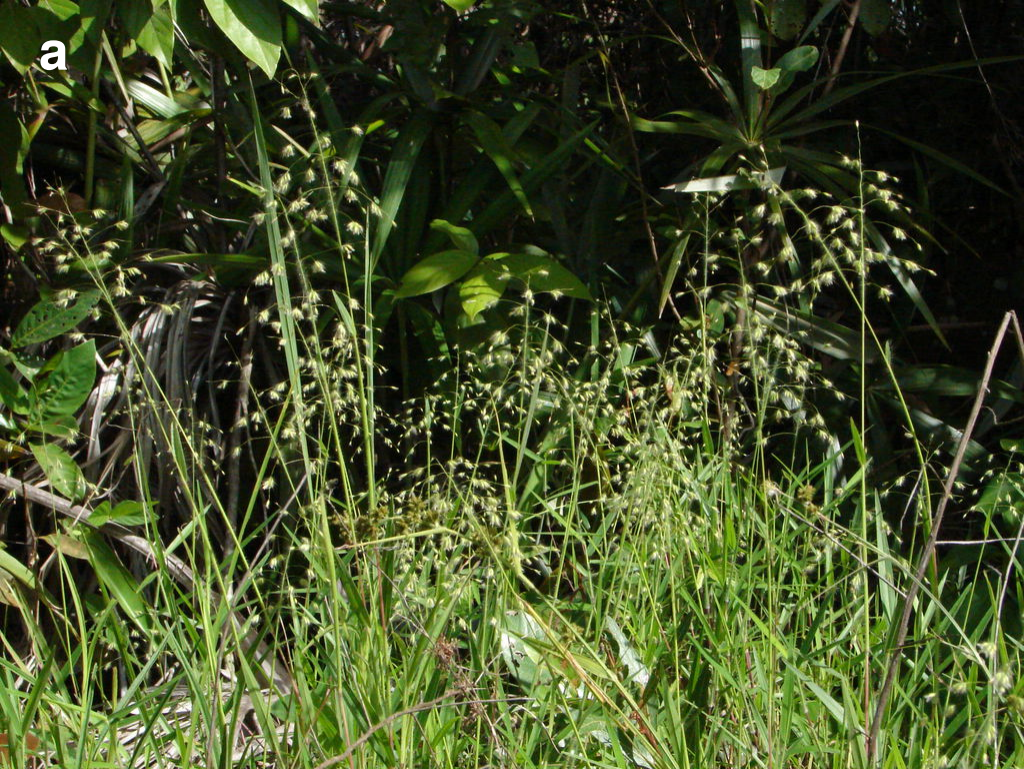
habit

**Figure 3b.**
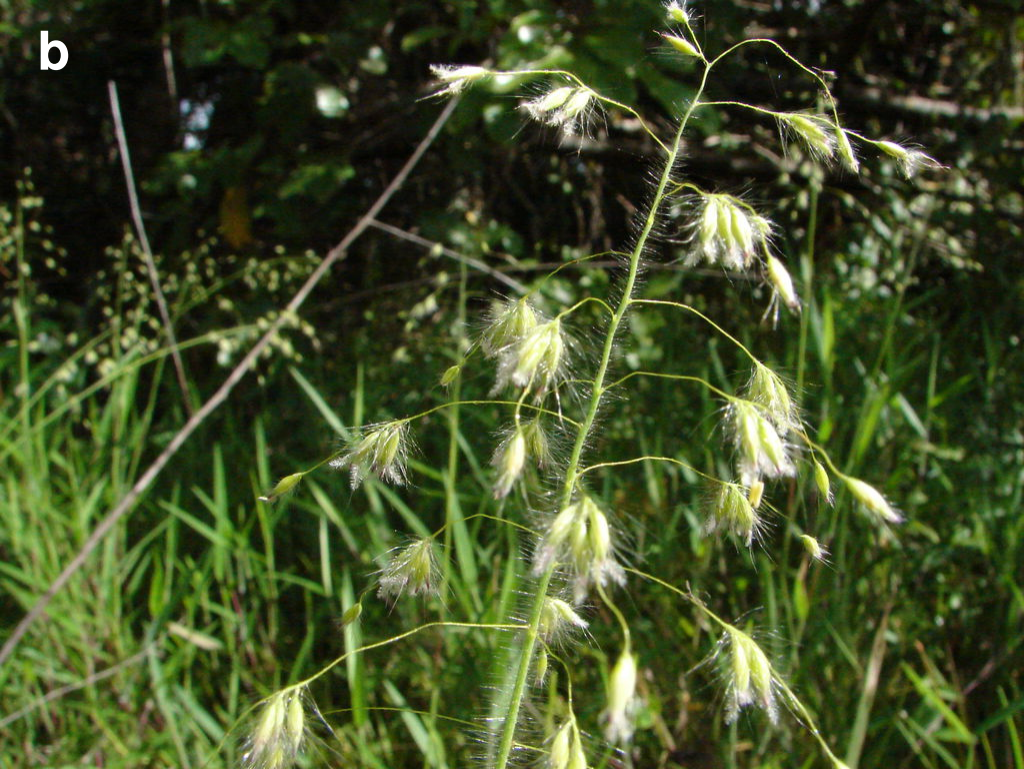
inflorescence

**Figure 4a.**
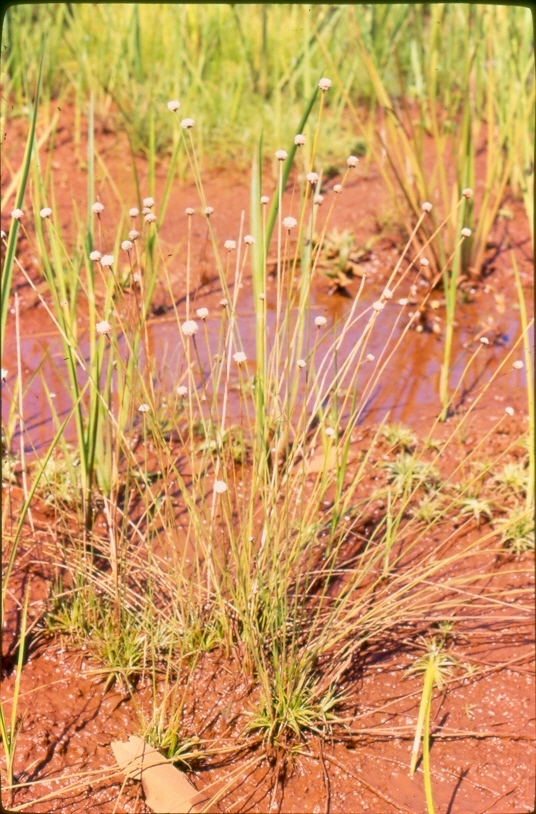
habit

**Figure 4b.**
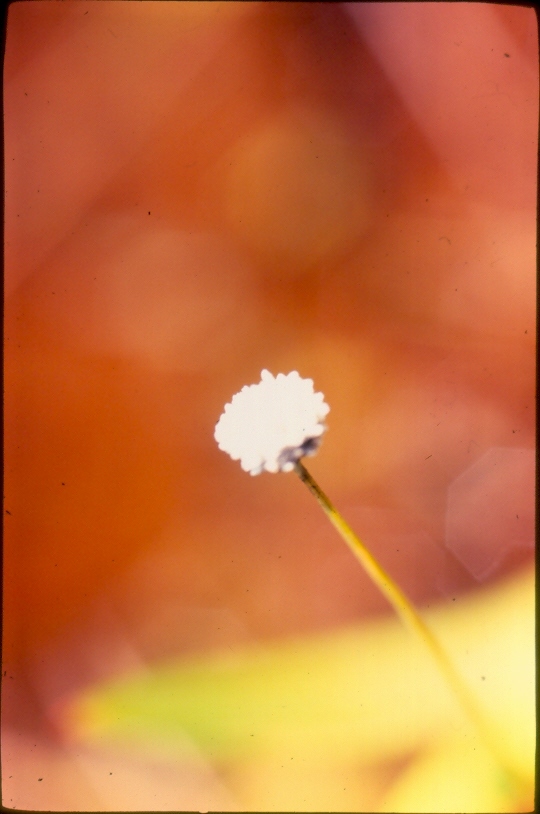
Inflorescence

**Figure 5a.**
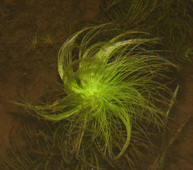
habit

**Figure 5b.**
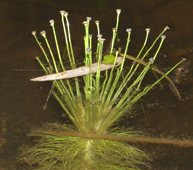
inflorescence

**Table 1.** Checklist of hydrophylous Monocots from the Araguaia River basin. Species found in consulted herbaria (Sh); species found in *Species* Link (Ss); new occurrences to Araguaia River basin (NoA); new occurrences to Cerrado (NoC); geographic distribution (Gd): A Americas, B Brazil, W Worldwide; Life forms (Lf): He Helophytes, Em emergent, Ff Floating-fixed, Fr floating-free, Sf submerged-fixed.

**Family**	**Species**	**Lf**	**Gd**	**NoA**	**NoC**	**Sh**	**Ss**
Alismataceae	*Echinodorus longipetalus* Mich.	Em	A			+	+
	*E. subalatus* (Mart. ex Schult. f.) Griseb.	Em	A			+	
	*Helanthium bolivianum* (Rusby) Lehtonen & Myllys	Em /Sf	A			+	+
	*H. tenellum* (Mart. ex Schult.f.) J.G.Sm.	Em /Sf	A			+	
	*Hydrocleys nymphoides* (Willd.) Buchenau	Ff	A			+	
	*H. parviflora* Seub.	Ff	A			+	
	*Limnocharis laforestii* Griseb.	Em	A			+	
	*Sagittaria guayanensis* Kunth	Fr	W			+	+
	*S. rhombifolia* Cham.	Em	A			+	+
Araceae	*Anthurium lindmanianum* Engl.	He	B	+		+	+
	*Dieffenbachia aglaonematifolia* Engl.	He	A	+			+
	*D. seguine* (Jacq.) Schott	He	A	+		+	
	*Gearum brasiliense* N.E.Br.	He	B				+
	*Philodendron imbe* Schott	He	B	+	+		+
	*P. quinquenervium* Miq.	He	A	+		+	+
	*Urospatha sagittifolia* (Rudge) Schott	He	A			+	+
	*Xanthosoma striatipes* (Kunth & C.D.Bouché) Madison	He	A	+		+	+
Arecaceae	*Desmoncus polyacanthos* Mart.	He	A	+			+
	*Syagrus petraea* (Mart.) Becc.	He	A	+			+
Burmanniaceae	*Apteria aphylla* (Nutt.) Barnhart ex Small	He	A			+	
	*Burmannia capitata* (Walter ex J.F.Gmel.) Mart.	He	A	+		+	+
	*B. flava* Mart.	He	A			+	+
Cannaceae	*Canna indica* L.	He	A				+
Commelinaceae	*Commelina erecta* L.	He	W				+
	*Commelina diffusa* Burm. f.	He	W	+		+	
	*C. obliqua* Vahl	He	A			+	
Costaceae	*Costus spiralis* (Jacq.) Roscoe	Em	A			+	+
Cyperaceae	*Ascolepis brasiliensis* (Kunth) Benth. ex C.B. Clarke	Em	W			+	+
	*Bulbostylis stenocarpa* Kük.	He	A	+			+
	*Calyptrocarya glomerulata* (Brongn.) Urb.	Em	A			+	+
	*Cyperus diffusus* Vahl	He	W	+			+
	*C. digitatus* Roxb.	Em	W			+	
	*C. giganteus* Vahl	Em	A	+			+
	*C. haspan* L.	Em	W			+	+
	*C. odoratus* L.	He	W			+	+
	*C. pohlii* (Nees) Steud.	Em	A	+			+
	*C. schomburgkianus* Nees	He	A	+			+
	*C. sphacelatus* Rottb.	He	W	+			+
	*C. tenuispica* Steud.	He	W	+			+
	*Diplacrum capitatum* (Willd.) Boeck.	Em	A	+			+
	*Eleocharis acutangula* (Roxb.) Schult.	Em	W			+	
	*E. capillacea* Kunth	Em/Sf	A			+	
	*E. filiculmis* Kunth	Em	A			+	+
	*E. interstincta* (Vahl) Roem. & Schult.	Em	A			+	
	E. minima var. minima Kunth	Em/Sf	A	+			+
	E. minima var. bicolor (Chapm.) Svenson	Em/Sf	A			+	
	*E. nana* Kunth	Em	A			+	
	*E. nudipes* (Kunth) Palla	Em	A			+	
	*E. plicarhachis* (Griseb.) Svenson	Em	A			+	
	*E. retroflexa* (Poir.) Urb.	Em	W			+	
	*E. sellowiana* Kunth	Em	A			+	
	*Fimbristylis aestivalis* (Retz.) Vahl	Em	W			+	+
	*F. dichotoma* (L.) Vahl	Em	W			+	
	*F. quinquangularis* (Vahl) Kunth	Em	W			+	
	*Fuirena umbellata* Rottb.	Em	W			+	+
	*Kyllinga brevifolia* Rottb.	Em	W	+			+
	*K. odorata* Vahl	Em	W				+
	*K. vaginata* Lam	He	W	+			+
	*Lipocarpha chinensis* (Osbeck) J.Kern	Em	W			+	+
	*Pycreus lanceolatus* (Poir.) C.B.Clark	He	W			+	+
	*P. unioloides* (R.Br.) Urb.	He	W			+	+
	*Rhynchospora armerioides* J.Presl & C.Presl.	He	A				+
	*R. barbata* (Vahl) Kunth	He	A			+	+
	*R. brevirostris* Griseb.	He	W			+	+
	*R. corymbosa* (L.) Britton	Em	W			+	
	*R. divaricata* (Ham.) M.T.Strong	Em	A	+			+
	*R. exaltata* Kunth	He	A				+
	*R. filiformis* Vahl	He	A	+			+
	*R. globosa* (Kunth) Roem. & Schult.	Em	A			+	+
	*R. hassleri* C.B.Clarke	Em	A	+			+
	*R. rugosa* (Vahl) Gale	He	W			+	+
	*R. tenuis* Link.	Em	A				+
	*R. trispicata* (Nees) Schrad. ex Steud.	Em	A			+	+
	*R. aff. unisetosa* T. Koyama	He	A	+	+		+
	*R. velutina* (Kunth) Boeck.	Em	A			+	
	*Scleria gaertneri* Raddi	He	W			+	+
	*S. microcarpa* Nees ex Kunth	Em	A	+			+
	*S. mitis* P.J.Bergius	He	A			+	
Eriocaulaceae	*E. alto-gibbo* sum Ruhland	Em	B	+		+	
	*Eriocaulon araguaiense* A.Oliveira & C.P.Bove	Em	B			+	
	*E. cylindratum* A.Oliveira & C.P.Bove	Em	B			+	
	*E. epapillosum* Ruhland	He	B	+		+	
	*E. humboldtii* Kunth	Em	B				+
	*E. setaceum* L.	Em/Sf	B			+	
	*Comanthera*. *xeranthemoides* (Bong.) L.R. Parra & Giul.	He	B			+	
	*Paepalanthus viridis* Körn.	He	B	+		+	
	*Syngonanthus caulescens* (Poir.) Ruhland	He	B			+	+
	*S. densiflorus* (Körn.) Ruhland	Em	A				+
	*S. gracilis* (Bong.) Ruhland	He	B			+	
	*S. helminthorrihizus* (Mart.) Ruhland	He	B	+		+	
	*S. humboldtii* (Kunth) Ruhland	Em	A	+			+
	*S. longipes* Gleason	Em	A				+
	*S. nitens* (Bong.) Ruhland	He	A				+
	*Tonina fluviatilis* Aubl.	Sf	A				+
Heliconiaceae	*Heliconia psittacorum* L.f.	He	B			+	+
Hydrocharitaceae	*Elodea granatensis* Humb. & Bonpl.	Sf	A	+		+	
	*Egeria heterostemon* S.Koehler & C.P.Bove	Sf	B	+		+	
	*Najas affinis* Rendle	Fr/Ff	W	+		+	
	*Ottelia brasiliensis* (Planch.) Walp.	Sf	A	+		+	+
Marantaceae	*Thalia geniculata* L.	He	W	+		+	+
Mayacaceae	*Mayaca fluviatilis* Aubl.	He	A	+		+	+
	*M. longipes* Mart. ex Seub.	He	A	+		+	
	*M. madida* (Vell.) Stellfeld	He	A	+		+	
Orchidaceae	*Bletia catenulata* Ruiz & Pav.	He	A	+			+
	*Epidendrum densiflorum* Hook.	He	A	+			+
	*Habenaria macilenta* (Lindl. ex Benth.) Rchb.f.	He	A	+			+
	*H. orchiocalcar* Hoehne	He	B	+			+
	*H. spathulifera* Cogn.	He	A	+			+
Poaceae	*Acroceras fluminense* (Hack.) Zuloaga & Morrone	He	A	+			+
	*Andropogon lateralis* Nees	He	A	+			+
	*A. leucostachyus* Kunth	He	A	+			+
	*Digitaria ciliaris* (Retz.) Koeler	He	W	+		+	+
	*D. filiformis* (L.) Kaler	He	A			+	
	*Echinochloa colona* (L.) Link	He	W	+		+	+
	*E. crus-galli* (L.) P.Beauv.	He	W	+			+
	*Eleusine indica* (L.) Gaertn.	He	W	+			+
	*Eragrostis japonica* (Thunb.) Trin.	He	W	+		+	
	*E. rufescens* Schult.	He	A	+			+
	*Eriochrysis cayennensis* P. Beauv.	He	A	+			+
	*Hymenachne amplexicaulis* (Rudge) Nees	He	A	+		+	+
	*Mesosetum cf. loliiforme* (Steud.) Hitchc.	He	A	+			+
	*Ichnanthus procurrens* (Nees ex Trin.) Swallen	He	A	+		+	+
	*Isachne polygonoides* (Lam.) Döll	He	W	+		+	+
	*Luziola peruviana* Juss ex J.F. Gmel.	He	W	+		+	
	*Panicum cyanescens* Nees ex Trin.	He	A	+		+	+
	*P. discrepans* Döll	He	A	+		+	
	*P. latissimum* J.G.Mikan ex Trin.	He	B	+	+		+
	*P. parvifolium* Lam.	He	W	+		+	+
	*P. repens* Berg.	He	W	+		+	
	*P. schwackeanum* Mez.	He	A	+		+	
	*P. stenodes* Griseb.	He	A	+			+
	*P. trichoides* Sw.	He	A	+			+
	*Paspalum cinerascens* (Döll) A.G.Burm. & M.Bastos	He	A	+			+
	*P. convexum* Humb. & Bonpl. ex Flüggé	He	A	+			+
	*P. gardnerianum* Nees	He	A	+			+
	*P. glaucescens* Hack.	He	A	+			+
	*P. maritimum* Trin.	He	A	+			+
	*P. marmoratum* Kuhlm.	He	A	+		+	
	*P. pictum* Eckm.	He	A	+		+	+
	*P. repens* Berg.	He	A	+		+	+
	*P. trichotomum* Hack.	He	B	+			+
	*Reimarochloa acuta* (Fluggé) Hitchc.	He	A	+		+	
	*Sacciolepis vilvoides* (Trin.) Chase	He	A	+			+
	*Setaria parviflora* (Poir.) M.Kerguelen	He	A	+			+
	*Steinchisma decipiens* (Nees ex Trin.) W.V.Br.	Em	A	+		+	
	*S. laxum* (Sw.) Zuloaga	He	W	+		+	
	*Thrasya thrasyoides* (Trin.) Chase	He	A	+			+
Pontederiaceae	*Eichhornia azurea* (Sw.) Kunth	Ff	A	+		+	
	*E. crassipes* (Mart.) Solms	Fr/ Ff	A			+	
	*E. diversifolia* (Vahl) Urb.	Ff	A			+	
	*Pontederia parviflora* Alexander	Ff	A	+		+	
	*P. subovata* (Seub.) Lowden	Ff	A			+	
Rapateaceae	*Cephalostemon gracilis* (Poepp. & Endl.) R.H.Schomb.	Em	B	+		+	+
Typhaceae	*Typha domingensis* Pers.	He	W	+		+	+
Xyridaceae	*Abolboda pulchella* Bonpl.	He	A	+			+
	*Xyris caroliniana* Walter	He	A	+			+
	*X. fragilis* Kral & Lor.B.Sm.	Em	B	+			+
	*X. jupicai* Rich.	He	A	+		+	+
	*X. savanensis* Miq.	He	A	+		+	+
	*X. tenella* Kunth	He	A	+		+	
	*X. tortula* Mart.	He	A	+			+
Zingiberaceae	*Hedychium coronarium* J.König	He	W	+		+	
